# Amplification-Free Quantification of Endogenous Mitochondrial DNA Copy Number Using Solid-State Nanopores

**DOI:** 10.1021/acsnano.5c00732

**Published:** 2025-03-13

**Authors:** Sohini Pal, Diana Huttner, Navneet C. Verma, Talya Nemirovsky, Oren Ziv, Noa Sher, Natalie Yivgi-Ohana, Amit Meller

**Affiliations:** † Faculty of Biomedical Engineering, Technion -IIT, Haifa 3200003, Israel; ‡ Minovia Therapeutics Ltd., Tirat Carmel 3902603, Israel; § Russell Berrie Nanotechnology Institute, Technion -IIT, Haifa 3200003, Israel

**Keywords:** mitochondrial DNA, solid-state nanopores, single-molecule analysis, amplification-free quantification, purification-free assay, TFAM, electro-optical nanopore sensing

## Abstract

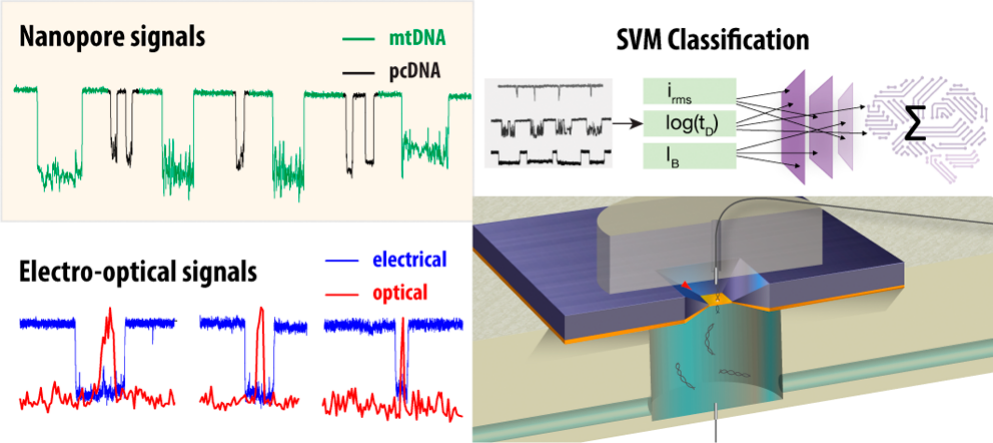

Mitochondrial DNA (mtDNA) quantification is crucial in understanding mitochondrial dysfunction, which is linked to a variety of diseases, including cancer and neurodegenerative disorders. Traditional methods often rely on amplification-based techniques, which can introduce bias and lack the precision needed for clinical diagnostics. Solid-state nanopores, an emerging biosensing platform, have the advantage of offering single-molecule and label-free approaches by enabling the direct counting of DNA molecules without amplification. The ion-current signatures obtained from each DNA molecule contain rich information on the molecules’ lengths and origin. In this study, we present an amplification-free method for mtDNA quantification using solid-state nanopores and machine learning. Intriguingly, we find that native (unamplified) mtDNA translocations harbor structurally distinctive features that can be exploited to specifically detect and quantify mtDNA copies over the background of genomic DNA fragments. By combining selective degradation of linear genomic DNA (gDNA) via exonuclease V with a support vector machine (SVM)-based model, we isolate and quantify mtDNA directly from biological samples. We validate our method using plasmids or isolated mtDNAs by spiking in predetermined quantities. We then quantify endogenous mtDNAs in a cancer cell line and in blood cells and compare our results with qPCR-based quantification of the mtDNA/nuclear DNA ratios. To elucidate the source of the ion-current signatures from the native mtDNA molecules, we perform synchronous electro-optical sensing of mtDNAs during passage through the nanopore after NHS ester reaction with fluorophore compounds. Our results show correlated electro-optical events, indicating that the mtDNA is complexed with packaging proteins. Our assay is robust, with a high classification accuracy and is capable of detecting mtDNA at picomolar levels, making it suitable for low-abundance samples. This technique requires minimal sample preparation and eliminates the need for amplification or purification steps. The developed approach has significant potential for point-of-care applications, offering a low-cost and scalable solution for accurate mtDNA quantification in clinical settings.

Mitochondria are cytoplasmic organelles essential for the efficient production of energy in mammalian cells, containing their own independent genome, a circular 16,659 base pair (bp) long double-stranded DNA (dsDNA) in humans.^1^ Mitochondrial DNA (mtDNA) exists in nearly every cell in a wide range of abundance, ranging from hundreds to tens of thousands of copies, and if compared to the nuclear diploid DNA, which consists of ∼6 billion bp, it can be estimated to represent only a few percent of the total DNA mass in high copy number cells.^2^ mtDNA encodes 37 genes, 13 of which are proteins related to oxidative phosphorylation.^1^ Mitochondrial dysfunction, as well as variations in mtDNA copy number, is related to altered energy homeostasis, manifesting in aging, neurodegenerative disorders, and cancer.^1^ The “gold standard” method for mtDNA copy number quantification, qPCR, heavily relies on the amplification of mitochondrial-encoded genes and the ratio to amplified nuclear-encoded genes. Bias, however, may arise due to inherent differences in the amplification efficiency or off-target amplification,^3^ and is dependent on full sequence identity between primers and template. Recent advances in nanopore-based technology allow high-throughput sequencing of long reads and have been shown to be especially useful for mtDNA mapping using genome-wide sequencing.^3,4^ However, these single-molecule mtDNA sequencing methods require efficient mtDNA linearization, library preparation, and purification steps, and often include strategies to enrich for mtDNA copies to overcome the inherent poor coverage of mtDNA reads.^4,5^ Additionally, mechanical stress applied during sample manipulation limits the average read length sequenced^4^ and the inherent nanopore noisiness of the sequence reads^5^ challenges *de novo* assemblies devoid of errors. This latter challenge is partially solved with improved analysis workflows.^6^ Nevertheless, these limitations complicate the usage of these single-molecule sequencing methods for reliable mtDNA copy number quantification.^2−4^

Solid-state nanopore (ssNP) is an emerging biosensing platform that offers single-molecule and label-free counting of DNA molecules without amplification,^7^ while potentially permitting high sensitivity and high throughput.^8−12^ This technology involves passing DNA molecules, one by one, through nanoscale pores formed in a silicon nitride membrane or through pulled glass pipettes while measuring changes in ionic current upon voltage application across the pore. The ion-current signatures obtained from each DNA molecule contain information on the molecules’ length and their structure.^13−17^ Of all nanopore kinds, ssNPs are particularly useful for the analysis of dsDNA molecules due to their extreme sensitivity^18^ and the fact that nanopore size can be tuned to fit single-file passages of the dsDNA biopolymer (typical cross-section ∼2.5 nm).^19−21^ However, to adapt the ssNP technology toward low copy-number *endogenous* mtDNA quantification, a few outstanding challenges must be met. Specifically, preventing ssNP clogging and false events readout by biosample background molecules, and controlled attenuation of the vast genomic DNA (gDNA) fragments, without diminishing the mtDNA copies in the sample. Notably, the use of purification kits may achieve the former but are prone to introduce significant and highly variable losses to the mtDNA and hence bias the results. These obstacles must be mitigated to advance the NPs sensing closer toward rapid and efficient molecular diagnostics from heterogeneous and complex biosamples, with seamless and broadly accessible translation for point-of-care environments.^17,22−26^

To address these challenges, we present a straightforward, amplification-free, purely enzymatic assay coupled with ssNP sensing that permits robust and accurate mtDNA copy number quantification from biological or clinical samples. Our method is based on two key discoveries: (i) unlike synthetic (i.e., PCR-amplified) dsDNA, bacterial-isolated plasmids, or exonuclease V fragmented gDNA, the time-dependent nanopore signals from biological mtDNAs exhibit distinctive features, which can be used to isolate them from other molecules using a Support Vector Machine (SVM) Model; (ii) a selective degradation of only linear gDNA using ATP-regenerated exonuclease V activity to reach a steady state in fragmented gDNA baseline population that is roughly ∼10-fold greater than the mtDNA copy number. Combining these two developments, we realized an amplification-free method for fast digital counting of the endogenous mtDNA to the fragmented gDNA ratios from cancer or blood cells. Importantly, as our method does not involve centrifugation or purification stages, it is readily translatable to point-of-care applications in clinics, hospitals, and homes.

## Results and Discussion

### Native mtDNA Molecules Produce Characteristic Ion-Current Signatures in Solid-State Nanopores

**Figure 1:**
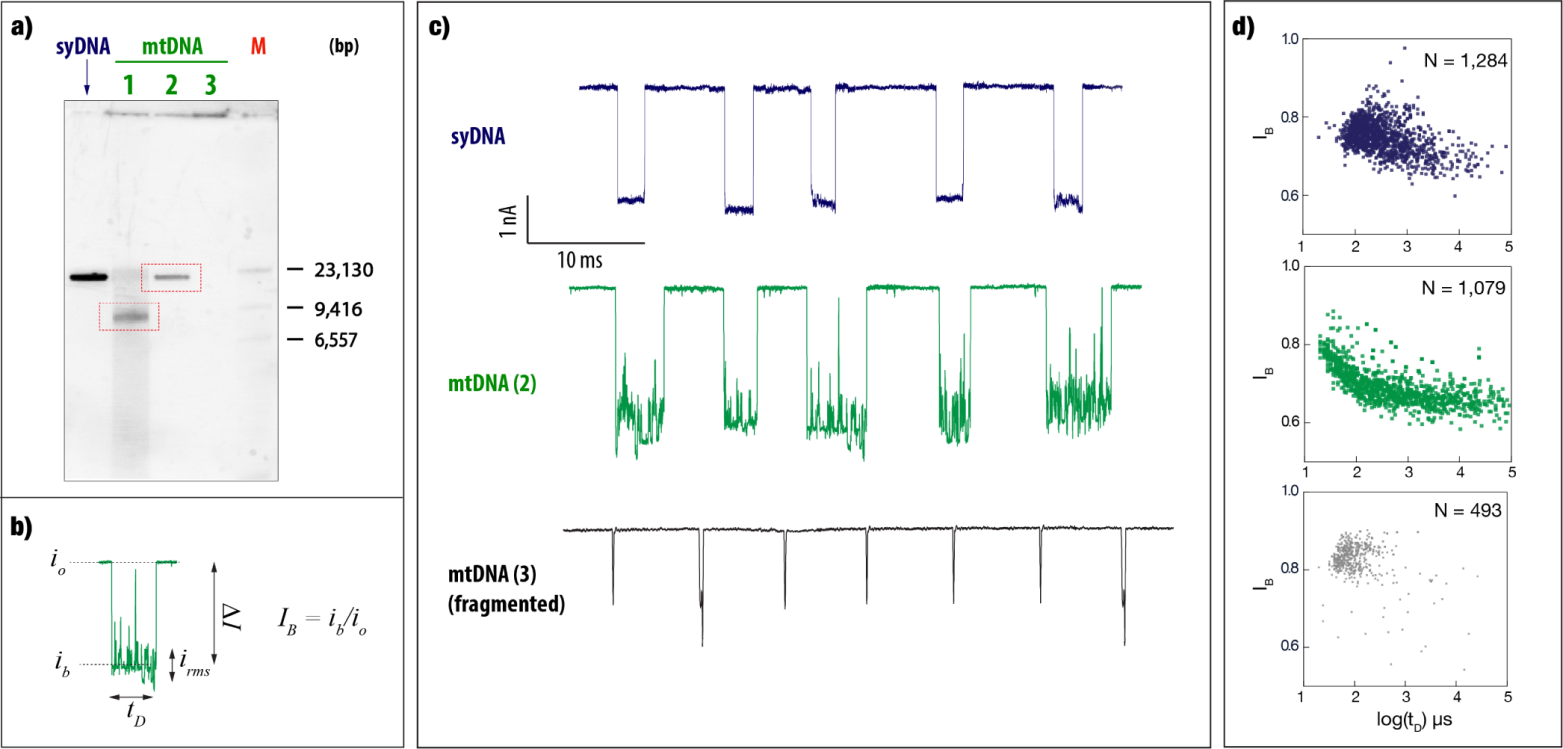
Linearized native mitochondrial DNA (mtDNA) shows distinctive ion-current signature. (a) 17,000 bp DNA (“syDNA”) was separated on an agarose gel along mtDNA (**1**) extracted from isolated mitochondria of human placenta, AflII-linearized mtDNA (**2**), and AflII-linearized mtDNA, which was subjected to fragmentation with AluI and *Hae*III restriction enzymes (**3**). λ DNA/*Hin*dIII was used as a molecular weight marker (**M**). b) Ion current trace showing the key parameters *i*_o_ (open pore current), *i*_b_ (blockage current), *t*_D_ (dwell time), Δ*I* (blockage amplitude), *i*_rms_ (RMS of blockage level), and fractional blockage current *I*_B_ = *i*_b_/*i*_o_. (c) Representative ion current signatures for syDNA, mtDNA, and fragmented mtDNA show distinct levels of current blockage. syDNA displays well-defined linear blockades (*i*_rms_ = 0.16 ± 0.04 nA), while linearized mtDNA shows irregular, complex blockades with ∼6-fold higher levels (*i*_rms_ = 1.1 ± 0.8 nA). Enzymatically fragmented mtDNA results in significantly shorter dwell times (typically <100 μs). (d) Scatter plots for syDNA (top), linearized mtDNA (middle), and fragmented mtDNA (bottom). The syDNA shows tighter clustering of events, while mtDNA has broader distribution patterns indicative of structural variability. Fragmented mtDNA shows a significant shift of *I*_B_ and *t*_D_ to lower values.

To establish the characteristic signals of native mtDNAs, we analyzed DNA molecules extracted from isolated mitochondria of human placenta (Methods). Mitochondrial DNA was found to remain in its supercoiled, natively looped state, as evidenced by agarose gel electrophoresis (Figure 1a (**1**)), exhibiting a broad smear with a noticeably darker band next to a ∼10 kbp DNA marker. Upon treatment of the mtDNA molecules with the restriction enzyme AflII (or alternatively with PvuII, Figure S1), having a unique cleavage site on human mtDNA, the expected uniform band of the linearized molecule around 17 kbp appears, as shown in Figure 1a (**2**). This band runs at a location indistinguishable from that of the reference standard, purified synthetic dsDNA molecules of uniform length of 17,000 bp (“NoLimits” DNA, ThermoFisher, syDNA). When the linearized mtDNAs were further subjected to the frequent four-nucleotide cutters AluI and *Hae*III, several shorter DNA fragments were produced, which were undetected by SYBR Gold staining (Figure 1a (**3**)).

In contrast to the gel analysis, the ssNP analysis of the sample produced highly distinctive signals, allowing us to distinguish among mtDNA and syDNA molecules of essentially the same length with single-molecule resolution. Figure 1c shows typical events of syDNA, mtDNA after linearization, and mtDNA after linearization and fragmentation (top to bottom, respectively). The syDNA produced a relatively uniform distribution of events in terms of their fractional blockage current (*I*_B_) and translocation dwell time (*t*_D_) in agreement with previous studies using similar ssNP sizes to analyze dsDNA molecules.^27,28^ Unlike syDNAs, the linearized mtDNA molecules exhibited distinctive fluctuating blocked current state, as shown in the middle panel of Figure 1c (see also SI Movie 1 showing nanopore translocation recordings for the mtDNA-enriched total DNA sample). Additionally, the mtDNA events displayed broader and longer *t*_D_ distribution and deeper event amplitude (lower *I*_B_ values) as compared with the similar length syDNAs. This may indicate that the extracted mtDNA harbors bound proteins or other structural features that significantly alter both *t*_D_ and *I*_B_.^29^ After the fragmentation step with the restriction enzymes AluI and *Hae*III, the translocation events were significantly shorter and exhibited a smaller event amplitude, as expected.

To further demonstrate the significance of the appearance of these distinctive event features, we summarized the results of three nanopore experiments carried out for each one of these samples separately in the event diagrams shown in Figure 1d. Exponential tail fits yield characteristic translocation dwell times of 680 ± 40 μs, 1,280 ± 75 μs, and 57 ± 4 μs for the syDNA, mtDNA, and fragmented mtDNA, respectively. Importantly, the appearance of intensive ion-current fluctuations only for the intact linearized mtDNA molecules presents an opportunity for clean separation of the mtDNA events from other DNAs by the inclusion of information from the in-event current fluctuations. In this regard, the most straightforward statistical property of the in-event current is *i*_rms_ defined as the RMS value of the blocked current level *i*_b_ (Figure 1b). Notably, we find that there is more than a 6-fold difference between the mean values of the *i*_rms_ of the syDNA (0.16 ± 0.04 nA) and the mtDNA (1.1 ± 0.8 nA) calculated for the full data sets (over 1,000 events for each type).

**Figure 2:**
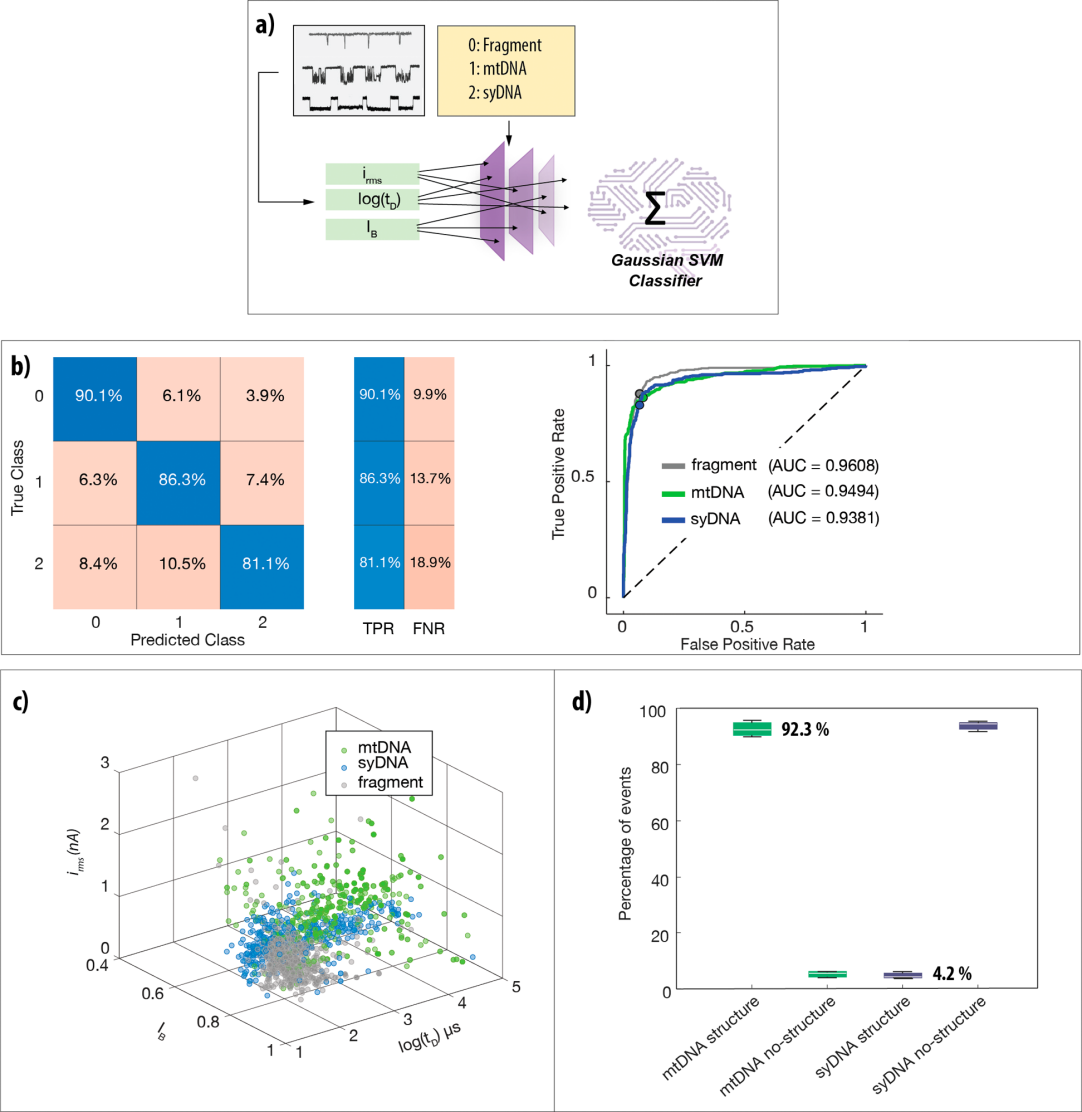
Statistical classification of mtDNA molecules showing high discrimination accuracy. (a) Schematic of the Gaussian support vector machine (SVM) classifier using the parameters RMS of current, dwell time, and relative blockage as its feature vectors. The classification system is set to identify three classes: fragments (Class 0), mtDNA (Class 1), and syDNA (Class 2). (b) Confusion matrix for the classifier shows an average prediction accuracy of 85.8% with a high accuracy of 90.1% for fragments (Class 0). True positive rates (TPR) or false negative rates (FNR) for each class are displayed on the right. Right panel: The AUC values for all classes average 0.95. (c) 3D scatter plot illustrating the separation of mtDNA, syDNA, and fragment events in the feature space of *I*_B_, log(*t*_D_), and *i*_rms_. mtDNA molecules (green) occupy a distinct cluster compared to syDNA (blue) and fragments (gray). (d) Bar plot showing the percentage of events exhibiting structural features. For mtDNA, around 92.3% of events displayed structural features, whereas only 4.2% of syDNA events exhibited structural complexity, highlighting the differences in their molecular architecture.

The results displayed in Figure 1 suggest that, in addition to the events’ dwell-time (*t*_D_) and fractional blockage current (*I*_B_), the events’ *i*_rms_ values are characteristic features. To that end, we evaluated the possibility of using a Support Vector Machine (SVM)-based model for event classification. The versatility of machine learning-based data analysis in solid-state nanopores has been demonstrated through its wide range of applications across various biological classes, including DNA,^30,31^ proteins,^32,33^ polysaccharides,^22,34^ and viruses.^35^ First, we trained an SVM model for separating three types of events (Figure 2), knowing that later reducing the possibility to only two event types (mtDNA and fragmented gDNA) would likely improve the classification accuracy. We implemented a multiclass SVM with a fine Gaussian kernel (Figure 2a) to differentiate the individual DNA events. The Gaussian kernel is particularly well-suited for cases where the data is not linearly separable, enabling it to capture complex relationships between its features. By integrating *I*_B_, *t*_D_, and *i*_rms_ values into the training set, we aimed to leverage these features for sample classification. To address potential class imbalance, we shuffled the data sets, which ensured equal representation of each sample, thereby preventing skewed model performance. A 5-fold cross-validation technique was applied, in which the data were divided into 5 sections with 20% of the data used as a test set, and the model was iteratively refined by evaluating its performance across different subsets of the data. By randomizing the data, we ensured that each fold in the cross-validation process contained a representative mix of the three classes, promoting better generalization during training and evaluation. The SVM achieved an average classification accuracy of 85.8%, with mtDNA fragments yielding a true positive rate of 90.1% (Figure 2b). The AUC values for all classes averaged 0.95, demonstrating the robustness and high classification performance of the SVM model. It should be noted that by incorporating current RMS values^36−39^ in the training protocol, our approach could classify the dsDNA samples of similar/same lengths (mtDNA and syDNA) with sufficient accuracy. Figure 2c illustrates the classification of the three types of DNA events: mtDNA fragments (gray), mtDNA (green), and syDNA (blue). Once the model had been thoroughly trained and validated, we applied it to examine event types in different experimental sets with mtDNA and syDNA (Figure 2d). Approximately 92.3% of mtDNA samples exhibited high *i*_rms_ values, indicative of inherent structural features, while only 4.2% of syDNA events demonstrated similar current signatures. These findings suggest that our SVM model is capable of reliably quantifying mtDNA in clinical settings despite challenges posed by small sample sizes.

### Biochemical Assay Verification Using Plasmids

**Figure 3:**
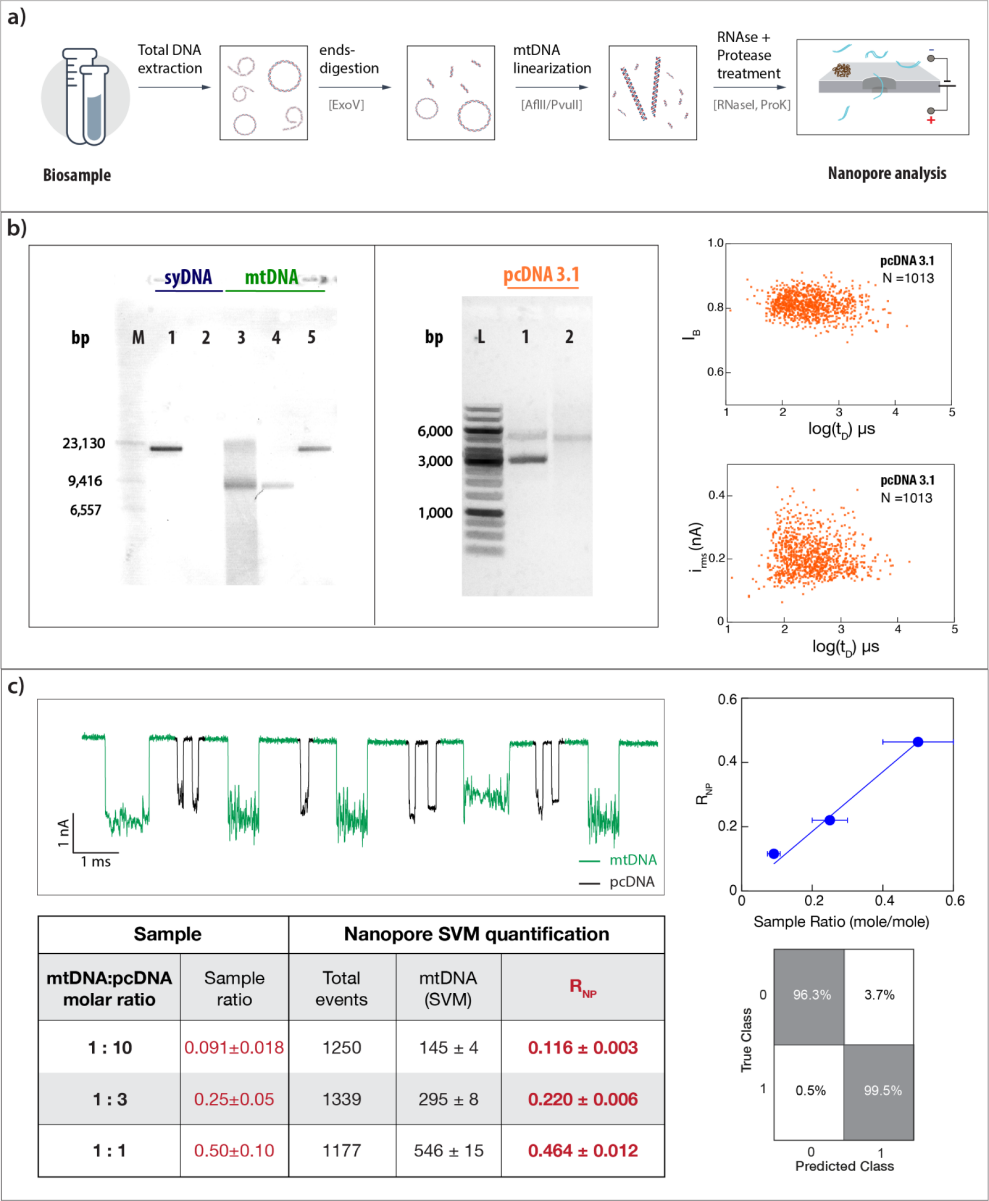
A biochemical nanopore assay for the analysis of mtDNA copy number from cells. (a) Workflow: total DNA is extracted from biosamples, followed by exonuclease treatment (ExoV) to selectively fragment linear gDNA into short pieces. Following ExoV inhibition, mtDNA is linearized with restriction enzymes (AflII or PvuII) and treated with ProK and RNaseI to degrade any leftover proteins and RNAs before nanopore analysis. (b) Assay validation using mtDNA extracted from isolated mitochondria (left panel). Gel lanes from left to right: λ DNA digested with *Hin*dIII DNA ladder (**M**), 17,000 bp DNA before (**1**) or after ExoV treatment (**2**), mtDNA before (**3**) or after ExoV (**4**) or the final AflII-linearized mtDNA sample (**5**). Assay validation was performed with plasmid DNA pcDNA3.1 (middle panel). Gel lanes from left to right: GeneRuler 1kb DNA ladder (**M**), pcDNA3.1 prepared without (**1**) or with (**2**) linearization. Corresponding scatter plots for linearized pcDNA translocations with *I*_B_, log(*t*_D_), and *i*_rms_ comparable to those of syDNA translocations show their structural integrity is preserved in the biochemical assay (right panel). (c) SVM model validation with mixtures of mtDNA and pcDNA at varying ratios. Representative ion current traces of the mixture, showing distinct differences in blockage levels, RMS, and dwell time for mtDNA and pcDNA (top panel). Table comparing the expected mtDNA ratios in the sample with SVM measurements from nanopore translocation events (bottom left). Confusion matrix of the SVM model with an average prediction accuracy of 97.9% (bottom right). Plot of the mtDNA:pcDNA molar ratio vs nanopore SVM based ratio (*R*_NP_) with a linear fit (right).

The ability to classify and count mtDNA molecules and gDNA fragments in a mixture is a mandatory requirement for the development of a nanopore quantification method for the mtDNA copy number. However, by itself, it is not sufficient. The expected extremely low copy number of mtDNA in biosamples, as compared with the high background of gDNA fragments, as well as other nucleic acids, could mask the mtDNA-specific signals and potentially block the pore. Molecular amplification and purification kits have been used to bypass these limitations but have their own disadvantages. Amplification methods, such as long-PCR, involve the synthesis of new sequences, which do not preserve the structural features of the original mtDNA molecules, as shown in Figure 1. Additionally, standard molecular purification procedures can bias the quantification accuracy, as they retain only a fraction of the desired molecules. To overcome this, we developed and validated a purely additive biochemical assay for the preparation of total DNA samples extracted from either cell lines or peripheral blood cells adequate for mtDNA nanopore analysis. To minimize sample losses and the risk of biasing the sample contents, we followed similar principles presented for PCR-free mRNA quantification using ssNPs,^40,41^ focusing on purely enzymatic treatments of the sample (Figure 3a), while avoiding any intervening purification, centrifugation, or filtration steps. To that end, we have carefully chosen enzymes that are active in a common buffer, which can be sequentially adjusted to accommodate the requirements of each enzymatic step. First, we use Exonuclease V (ExoV) to selectively degrade linear gDNAs while preserving the integrity of the circular mtDNA. To reach a steady state in gDNA fragmentation, we ran the ExoV digestion for an extended period of time by supplementing it with an ATP regeneration system (Figure S5). Following inactivation of ExoV, mtDNA is linearized using a site-specific restriction enzyme (AflII or PvuII) targeting a conserved region in the mtDNA that is unlikely to be mutated or deleted. Finally, background RNA molecules and the added enzymes are digested by RNase I and proteinase K (ProK), respectively, and the samples are subjected to ssNPs sensing assays.

Before utilizing our assay to analyze complex biosamples, we validated it in two setups: (i) First, we separately processed syDNA, mtDNA, or plasmids to validate each step of the process and confirm that no background “noise” molecules are produced during the biochemical process that could interfere with nanopore classification. The analyzed plasmids behaved as circular dsDNA models with known recognition sites for the three possible restriction enzymes chosen for mtDNA linearization (Figures S2 and S3). (ii) Second, we analyzed mixtures of mtDNAs and plasmids at known ratios to prove that the biochemical assay maintains the analyte ratio with minimal biases.

The plasmids used for both protocol validation setups were pcDNA3.1 (5,428 bp, Figure 3b) and pUC18 (2,686 bp, Figure S3B). After ExoV treatment and inactivation, the plasmids were linearized, and the mix was subjected to proteinase K (ProK) treatment to degrade the restriction enzymes with the expected results (Figure S3A). The middle panel in Figure 3b shows the plasmid samples before and after the full process separated on gel electrophoresis. Figure 3b (right panel) shows the nanopore events diagram for the pcDNA3.1 (*N* = 1,013). Figure S3C shows the nanopore events diagram for the pUC18 sample (*N* = 1186). Importantly, in both cases, we obtain a clean single population of events with the following characteristic dwell time, fractional blockage current, and *i*_rms_: 546 ± 30 μs, 0.81 ± 0.03, and 0.2 ± 0.07 nA for pcDNA3.1 and 320 ± 20 μs, 0.8 ± 0.03, and 0.14 ± 0.04 nA for pUC18, respectively.

Next, we applied the preparation procedure on mtDNA extracted from mitochondria isolated from human placenta. A synthetic dsDNA fragment of 17,000 bp NoLimits DNA (syDNA) was used as a control for the ExoV step. Figure 3b (left) shows the analysis of syDNA and purified mtDNA before (**1** and **3**) or after (**2** and **4**) ExoV treatments. As expected, linear syDNA is fully digested, whereas circular mtDNA is kept intact. Lane **5** shows the linearized mtDNA sample after the full process, namely: ExoV → AflII → RNaseI → PK. When comparing **3** and **4**, we also noticed that the isolated mitochondria mtDNA sample contains background free DNA (possibly gDNA bound to mitochondria) that disappears upon the extensive ExoV treatment, suggesting that our process potentially reduces background linear dsDNA.

To confirm that the linearized mtDNA translocation through the nanopore can be quantitatively distinguished from translocations of short dsDNA fragments in the same pore, we prepared mixtures of mtDNA (16,569 bp) and pcDNA3.1 (5,428 bp) at known ratios, subjecting them (as a mixture) to the full sample preparation followed by ssNPs analyses. Three mtDNA:pcDNA3.1 mixtures were prepared and quantified using Qubit (Methods): 1:10, 1:3, and 1:1. All samples were analyzed using the nanopore (Figure 3c), and classification of the events was performed as described in Figure 2. Similarly to the syDNA analyzed in Figure 1, the mean *i*_rms_ of the mtDNA was found to be much larger than the corresponding value for pcDNA3.1, significantly increasing the robustness of the classification. The top panel displays 12 representative events from the mixture experiments denoted accordingly (mtDNA in green and pcDNA3.1 in black). The table and graph display the nanopore-measured ratios (*R*_NP_) of mtDNA versus the prepared mixture mole ratios. As can be seen, our nanopore results reproduce precisely the expected values within the experimental pipetting errors. Notably, due to the digital counting nature of the nanopore analysis, its experimental error is significantly less than the bulk error estimation.

### Quantification of mtDNA from Human Cell Lines and Blood Cells

**Figure 4:**
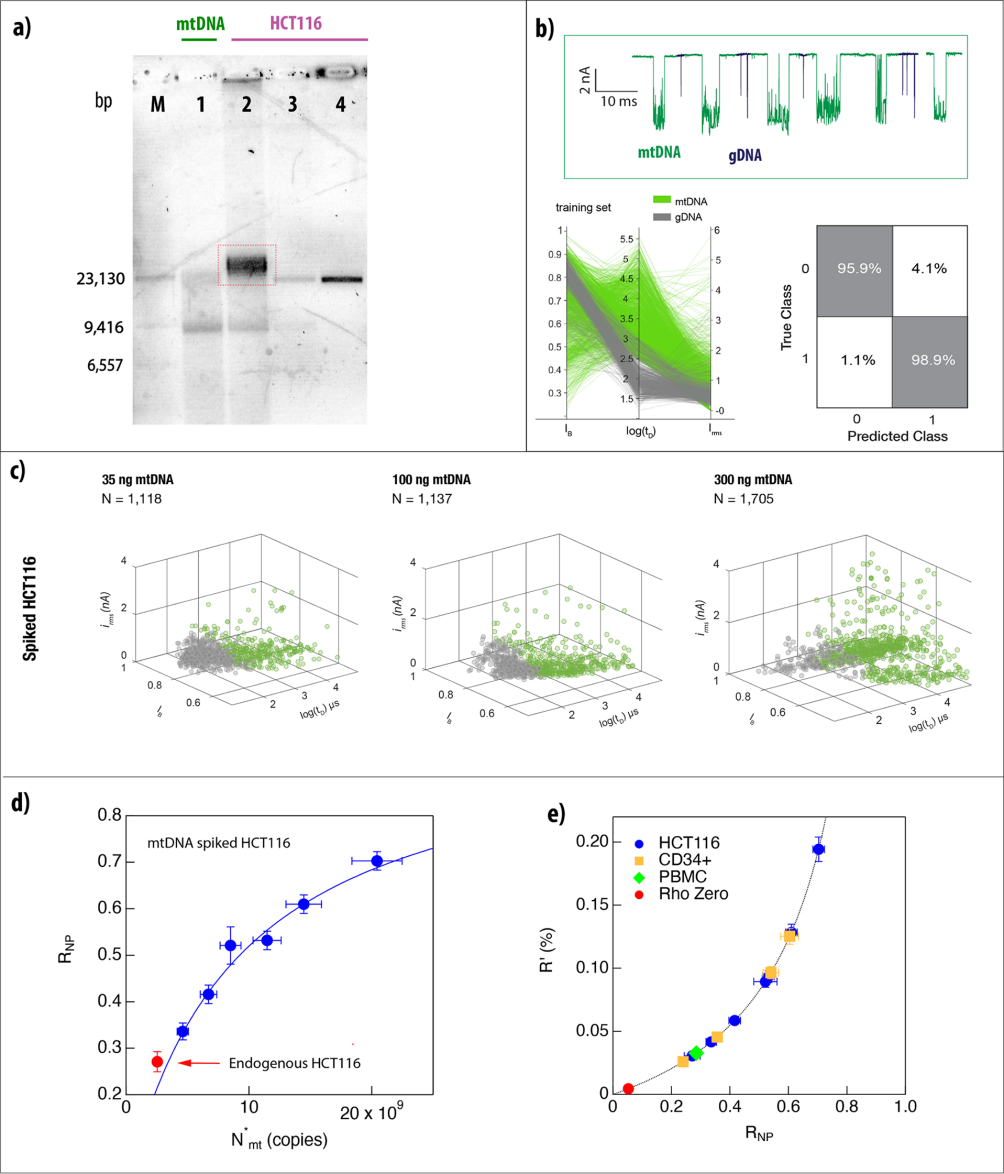
Amplification-free quantification of mtDNA from biosamples. (a) Gel showing different steps for total DNA preparations extracted from HCT116. Left to right: Digested λ DNA ladder (**M**), mtDNA extracted from isolated mitochondria (**1**), total DNA spiked with 100 ng of mtDNA (**2**), sample from **2** after ExoV treatment, inhibition, and linearization with AflII (**3**), sample from **2** after ExoV → AflII → RNaseI → ProK (**4**). (b) Representative nanopore signals showing the difference in current blockages for mtDNA (green) versus gDNA (black). (bottom left) Parallel coordinate plot of training data used to classify mtDNA and gDNA based on *I*_B_, log(*t*_D_), and *i*_rms_, indicating mtDNA and gDNA fragments form distinct clusters. (bottom right) Confusion matrix of the classification performance. The model achieves a high prediction accuracy with 95.9% for gDNA fragments (Class 0) and 98.9% for mtDNA (Class 1). (c) 3D scatter plots representing the classification of spike-in mtDNA in HCT116 cells at 35 ng (left), 100 ng (middle), and 300 ng (right). Each data set shows progressively increasing densities of mtDNA (green dots). d) The nanopore ratio (*R*_NP_) for spiked HCT116 DNA measured as in panel c vs the total mtDNA copy number fit by our model (Supporting Information eq 2). The red symbol indicates a measurement of the endogenous mtDNA (not included in the fit). (e) The expected mass/mass ratio of mtDNA to gDNA in percentage, based on eq 4 in the Supporting Information, of all the analyzed samples.

Next, we quantified the mtDNA copy number ratio to gDNA in three different total DNA samples extracted from human cells: (i) HCT116 colon cancer cell line, (ii) CD34^+^ hematopoietic stem and progenitor cells (HSPCs), and (iii) peripheral blood mononuclear cells (PBMCs). Additionally, we quantified the mtDNA-depleted cell line Rho Zero^42^ in order to establish a baseline for mtDNA detection. These cell types were chosen as they represent a range of mtDNA:gDNA ratios and demonstrate that the mtDNA signature is found across cells of different tissues. In addition, the use of PBMCs demonstrates that quantitation of copy numbers and the molecular signature will work on clinically relevant samples from peripheral blood. Before attempting to quantify the endogenous amounts of mtDNA, we calibrated our nanopore ratio quantification by “spiking in” bulk measured amounts of mtDNA (∼35 to 300 ng) from isolated mitochondria into HCT116 extracted DNA. During the preparation assay, samples were taken at each sequential step for agarose gel-based analysis (Figure 4a). Lane **1** shows the mtDNA extracted from the isolated mitochondria. Lanes **2**–**4** show the DNA extracted from the HCT116 cell lines and spiked with the isolated mtDNA, before the enzymatic treatment (**2**), after ExoV and AflII treatment (**3**), and finally following the RNaseI + ProK treatments (**4**). The heavier band in **2** (marked by a red square) corresponds to unfragmented gDNA, whereas the lighter band is the supercoiled mtDNA. At the final step of the protocol (**4**), a single band, corresponding in size to linearized mtDNA, is observed (as in Figure 1a). Notably, the ExoV treatment produces primarily mononucleotides or very short DNA fragments, which are not observable in either gel analysis or nanopore sensing. Tape station analysis (Figure S5), however, suggests that even after an extended ExoV reaction overnight, in the presence of an ATP regeneration system, a constant fraction of short DNA fragments remained. These short DNA fragments are readily detected in the nanopore as a stable gDNA fraction used as a proxy for the estimation of the total gDNA in each sample.

The samples were analyzed in the nanopore, and events were classified using the SVM model. In this case, only two types of events were observed: long translocations with large *i*_rms_ (corresponding to mtDNA) and very short events with low *i*_rms_ corresponding to the fragmented gDNA molecules. In Figure 4b, we show typical training and classification results coming from *N* = 1,079 (fragments) and N = 1,715 (mtDNA) events. The confusion matrix shows typical values of >96% of correct classification of the events. Typical raw translocation events are shown color-coded as before (mtDNA in green, gDNA in black). We repeated the sample preparation procedure and nanopore analysis using the same amount of total DNA extracted from the HCT116 cell lines spiked with six different amounts of the mtDNA (from 35 to 300 ng of mtDNA). All samples were analyzed by the ssNPs, and data were subject to the SVM model presented in Figure 2. Typical results are summarized in Figure 4d,e, and the full data sets are shown in Figures S7-S9 and Table S2. In these spike-in experiments, the adjusted gDNA copy number,  is kept constant (all came from the same HCT116 preparation), and measured amounts of mtDNAs to the endogenic amount are added to each sample. This permitted fitting of our data to a simple model (Supporting Information eq 2):
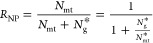
where  is a sum of the endogenic and spiked mtDNA. Leaving the endogenic mtDNA copy number and the gDNA as free parameters, we obtain =9.24 ± 0.75·10^9^ and *N*_mt_ = 2.53 ± 0.61·10^9^ copies. An independent nanopore measurement of the endogenous mtDNA:gDNA ratio in the HCT116 sample (red marker, not included in the fit) is consistent with our fit, further validating this simple prediction. A similar analysis was repeated with the total DNA extracted from CD34^+^ HSPCs. Three samples were spiked with 20, 40, and 80 ng of mtDNA from isolated mitochondria of human placenta, subjected to our biochemical protocol (Figure S7A), and analyzed using ssNPs.

To relate our measurements to the “gold-standard” qPCR ratio quantification,^43^ we co-analyzed PBMC cells by qPCR (Table S1) and the nanopore method (Figure S8). The nanopore and qPCR measured ratios of mtDNA:gDNA can be related via a single parameter as shown in Supporting Information eq 3. This allows us to evaluate the predicted mass:mass mtDNA to total DNA ratios in all biosamples as a function of the measured nanopore ratio *R*_NP_ (Figure 4e). Here, we also included the analysis of the mtDNA-depleted cell line Rho Zero, which may serve as a low-limit reference for the nanopore analysis, demonstrating its sensitivity. The full data set for this sample is shown in Figure S9. The HCT116 and CD34^+^ experiments suggest that the entire biochemical/nanopore workflow can be adapted to biological samples, yielding quantitative analysis of mtDNA to fragmented gDNA ratios.

### Electro-Optical Nanopore Measurements of NHS-Ester Labeled mtDNAs Suggest Complexation with Mitochondrial Proteins

**Figure 5:**
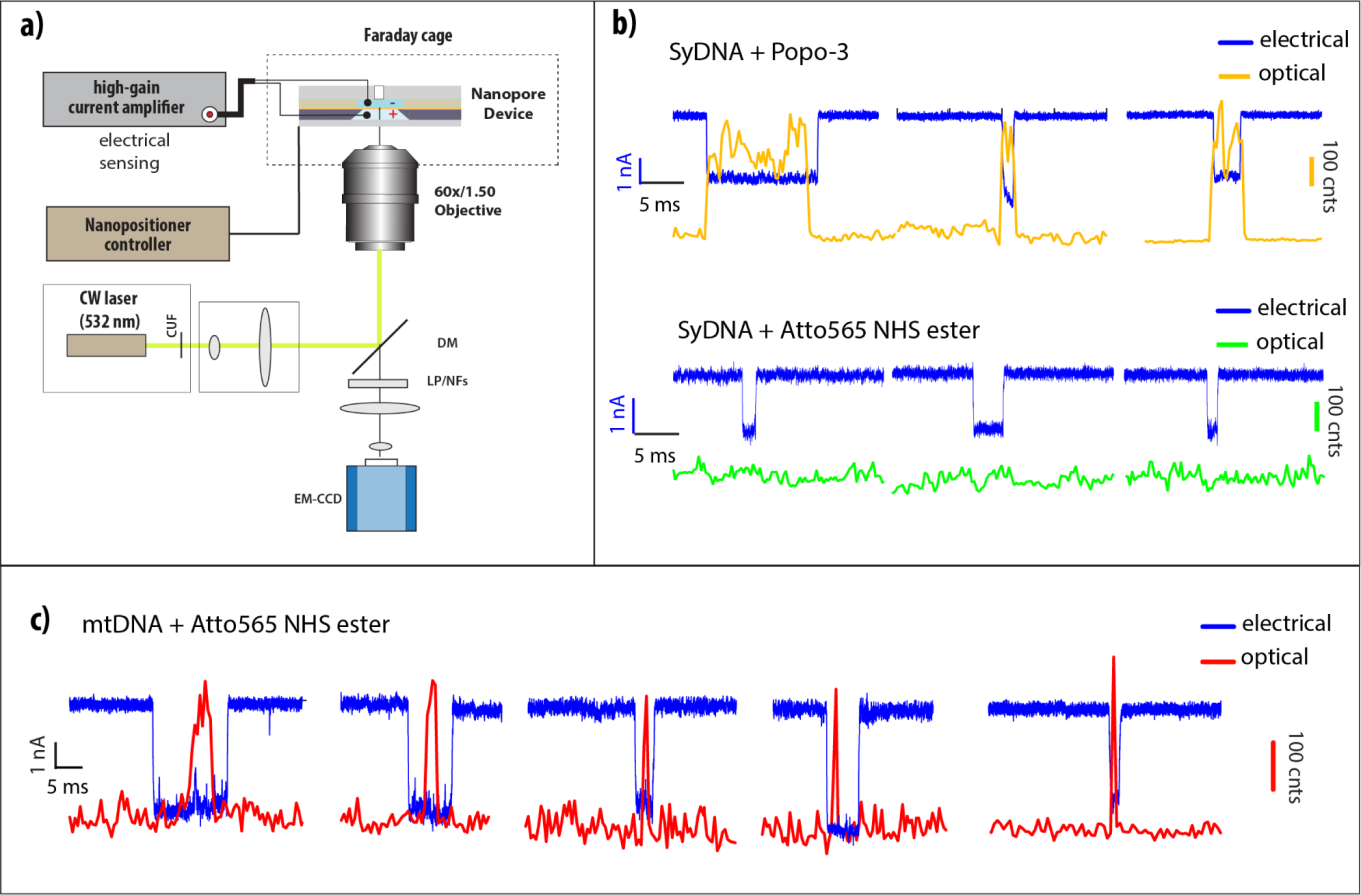
Electro-optical sensing of NHS ester labeled mtDNA suggests complexation with mitochondrial proteins. (a) Schematic layout of the Optipore setup used for synchronous electro-optical sensing of ssNPs. The nanopore devices are precisely positioned at the imaging spot of the setup using a piezoelectric nanopositioner, a custom optical cell that permits imaging of the chip with a high numerical aperture objective. The fluorescence light is collected via an EM-CCD device operated at 1,000 fps. (b) Control measurements: top panel displays typical electro-optical signals of Popo-3 stained 17 kbp DNA, demonstrating synchronous and sensitive sensing capabilities of the system. The lower panel shows typical electro-optical traces of Atto565 NHS ester labeled syDNA, that exhibit no correlated electro-optical signal, serving as negative control. (c) Typical electro-optical signals from Atto565 NHS ester mtDNA exhibit correlated optical photon bursts during the passage of the DNA through the pore. The NHS ester molecules interact with primary amines (likely lysine residues) indicating the complexation of the mtDNA with mitochondrial proteins.

DNA-bound proteins, such as transcription factors, have been shown to alter the nanopore ion-current signatures during their passage through the nanopore, leading to time-dependent current fluctuations.^29,44,45^ The magnitude of the ion-current blockage during translocation of DNA molecules through nanopores and its time dependency have often been interpreted in terms of the resistive pulsing model, suggesting that the amplitude of current blockage is, to a first-order approximation, proportional to the effective analyte’s cross-section.^46^ However, recent experiments and simulations of the steady-state ion current through charged nanopores suggest a more nuanced mechanism that also depends on the surfaces’ and molecules’ charges, particularly when the gap between the analyte and pore available for ions and water molecules is smaller than ∼1 nm.^47−50^ To elucidate the source of the observed ion current fluctuations and to relate it to the underlying mtDNA structure or bound proteins, we took advantage of a custom electro-optical sensing apparatus that permits synchronous sensing of fluorescence signals during the passage of individual DNA molecules through a solid-state NP (Figure 5a).^51^ mtDNA from isolated mitochondria of human placenta was first subjected to primary-amine specific labeling using NHS ester fluorophore (Atto565 NHS ester) at a concentration of ∼500 nM. The unconjugated dyes were consequently removed by dialyzing the sample using a 20 kDa cutoff membrane overnight against a clear Tris-acetate buffer, followed by the rest of our biochemical protocol as described in Figure 3a. Under the conditions used in our assay, the NHS ester is readily conjugated covalently to available primary amines, specifically targeting lysine residues, but has minimal or no interactions with nucleic acids. As a negative control, we identically processed syDNAs (Nolimits of 17 kbp).

Our results are presented in Figure 5b,c. The top panel in Figure 5b displays typical electro-optical events from syDNA that was prestained with the intercalating dye Popo-3, serving as a positive control. These results demonstrate the capability of our system to acquire synchronously the optical and electrical signals, with sub-millisecond accuracy.^52^ The lower panel in Figure 5b displays typical signals of the NHS ester-treated syDNA serving as a negative control. Here, no correlated electro-optical signal was observed, confirming that the NHS ester dyes do not interact with the syDNA, as expected. In contrast, when we evaluated the NHS ester-treated mtDNA, we observed an abundance of correlated electro-optical signals, indicating amine-specific conjugation to the mtDNA molecule complexes (Figure 5c). The optical photon bursts acquired by the EM-CCD were typically 300–500 counts per millisecond, about 10-fold higher than the typical RMS of the background level in our system. In some cases, the optical burst preceded the electrical translocation start, presumably due to the fact that dsDNA can remain in the pore vicinity for a brief time before its threading.^51^ In all experiments, we collected at least 300 events to ensure statistical robustness of the results. Since both the negative control (syDNA) and the mtDNA molecules underwent the same biochemical preparation (see Methods), the measurements suggest that mtDNA is likely complexed to mitochondrial proteins, which in turn give rise to the higher RMS in the ion current (Figure 1), distinguishing the native mtDNAs from syDNAs or plasmids.

## Conclusions

Imbalanced mitochondrial dynamics and mitochondrial dysfunction are associated with a range of diseases. Thus, mtDNA copy number, especially from attainable peripheral blood samples, is potentially an important disease biomarker. Current methodologies involve extensive amplification of short segments from mtDNA and gDNA and are prone to PCR-related biases. Here we demonstrate a robust and sensitive method for detecting and quantifying mtDNA using a solid-state nanopore system integrated with machine learning classification. By designing a carefully optimized biochemical preparation protocol that includes selective ExoV digestion and specific restriction enzyme cleavage, we achieved effective discrimination between mtDNA and gDNA fragments. It should be noted that multiple batches of the cell line samples and technical repetitions were prepared, yielding consistent ratios of mtDNA/gDNA as measured by the ssNPs. This further indicates that the biochemical assay reaches a steady-state level in terms of gDNA fragmentation. The reproducibility of our results highlights the robustness of our methodology, ensuring reliable mtDNA measurements that are critical for clinical applications. Incorporating Support Vector Machine (SVM) classification enhanced the detection and differentiation of mtDNA even in complex biological mixtures, enabling amplification-free detection at picomolar (pM) levels, streamlining workflows for real-time analysis.

A key finding of this study is that mtDNA ssNP translocations generate specific current fluctuations, which we leveraged for precise and sensitive endogenous mtDNA copy number identification and quantification using SVM. Importantly, these fluctuations must originate from inherent structures of mtDNA, which are lost in traditional amplification-based methods. Synchronous electro-optical measurements of mtDNA after reaction with the NHS ester fluorophore compounds (Figure 5) show correlated signals that indicate the possibility of stable complexation of the mtDNA with mitochondrial proteins. In the mitochondria, mtDNA forms nucleomimetic structures stabilized by interactions with the mitochondrial transcription factor A (TFAM) proteins, expressed at a high copy ratio to mtDNA.^53,54^ We therefore postulate that the ion-current fluctuations reflect regions of mtDNA-bound proteins, possibly TFAM, which are partially protected from ProK treatment. However, further studies are required to pinpoint the identity of the mtDNA-bound proteins. Nevertheless, our method already offers the opportunity to gain vital insights into the native form of mtDNA and can be further developed for the quantification of mtDNA/gDNA ratios in clinical samples.

In summary, we have presented a sensitive, reliable, and efficient approach to address the current limitations in mtDNA analysis using single-molecule counting principles. Notably, our biochemical process, as well as the ssNP device, are fully compatible with straightforward on-chip sample processing and single-molecule analysis, which can be further harnessed for enhanced diagnostics and research applications in mitochondrial biology and medicine. In addition to the direct classification of mtDNA copy numbers, future studies will also focus on further refining the ssNPs SVM model to quantify mitochondrial deletions, hence expanding the potential use of our method for heteroplasmy quantification in mitochondrial diseases.

## Materials and Methods

### Cell Lines, Plasmids, Clinical Samples, and Enzymes

The HCT116 colon cancer cell line (CCL-247) was purchased from the American Tissue Culture Collection (ATCC). CD34^+^ HSPCs (M34C-GCSF/MOZ/3) were purchased from Charles River Laboratories. The Rho Zero cell line A549p0#1a (catalog no. ESA113) was purchased from Kerafast. The peripheral blood sample was obtained from a healthy donor at the Hadassah Hospital, Jerusalem, Israel, for the LiquidBx consortium according to the regulations of the Clinical Research and Ethics Committee and the Helsinki Declaration of the World Medical Association (HMO 198–14). pUC18 and pcDNA3.1 plasmids were obtained from AddGene. A detailed description of the growth conditions of cell lines and plasmid isolation from *E. coli* can be found in the Supporting Information section. ExoV (RecBCD), AflII, PvuII, and *Bam*HI were purchased from New England Biolabs. RNase I and ProK were purchased from Thermo Fisher Scientific.

### Preparation of mtDNA from Isolated Mitochondria for Nanopore Analysis

Placenta was obtained from a healthy donor term in Carmel Hospital, Haifa, Israel, according to the regulations of the Clinical Research and Ethics Committee and the Helsinki Declaration of the World Medical Association (0185-18-CMC). Intact mitochondria were isolated by differential centrifugation using 250 mM sucrose buffer (pH 7.4). mtDNA was extracted using a Qiagen kit according to the manufacturer’s instructions. 200 ng of circular mtDNA was subjected to Exonuclease V (ExoV) treatment using 10 units at 37 °C for 1 h to remove any residual nuclear dsDNA. ExoV was inactivated for 30 min at 70 °C, followed by adjustment of the buffer composition and addition of 2 units of AflII for mtDNA linearization. Then, the sample was equally divided into two tubes. The first tube was supplemented with water, whereas the second tube was supplemented with AluI and *Hae*III (5 units of each) to achieve mtDNA fragmentation. Following incubation at 37 °C for 2 h, the enzymes were inhibited using 80 °C for 20 min. Subsequently, 0.8 units of ProK were added to each sample, and the samples were incubated for an additional 1 h at 37 °C.

### Preparation of Plasmid and mtDNA Mixtures for Method Validation

Isolated pcDNA3.1 plasmid or mtDNA, extracted from isolated mitochondria, were diluted in triplicates and quantified separately using the Qubit apparatus. Average molar concentrations were calculated from the measured concentrations (obtained in ng/μL), according to the theoretical size of each molecule, and then each molecule was serially diluted in sterile Milli-Q water. These stock dilutions were again measured using the Qubit to check that their concentrations were as expected. Then, these were used to mix mtDNA to pcDNA3.1 ratios of molar concentration, 1:1, 1:3, and 1:10, using 1 fmol of mtDNA in each case, and 1, 3, and 10 fmol of pcDNA3.1, respectively. Pipetting errors were estimated to be <20% in each case. The mixtures were subjected to ExoV treatment using 5 units at 37 °C for 1 h, then inactivated for 30 min at 70 °C. Two units of AflII were used for plasmid and mtDNA linearization at 37 °C for 2 h, followed by inactivation for 20 min at 65 °C. Finally, 0.8 units of ProK were added to each sample, and the samples were incubated for an additional 1 h at 37 °C. Complete linearization of the mixtures was confirmed by gel electrophoresis (data not shown). The samples were subjected to ssNPs analysis, followed by SVM classification.

### Preparation of Total DNA Samples for Nanopore Analysis

1 μg or 200 ng of total DNA samples, extracted either from HCT116 colon cancer cells or from human CD34^+^ cells, respectively, were supplemented with different amounts of exogenous circular mtDNA, extracted from isolated mitochondria of human placenta. For the endogenous mtDNA analysis, the same quantity of total DNA was used, without the addition of exogenous mtDNA. Similarly, total DNA was extracted from PBMCs of a healthy donor or from the mtDNA-depleted Rho Zero cell line. These samples were subjected to two rounds of addition of ExoV at 37 °C, each time using 20 units in the presence of a 1x ATP regeneration system (BML-EW9810–0100, ENZO Life Sciences). After inhibition of ExoV for 30 min at 70 °C, 4 units of AflII (or PvuII) were added, and the buffer composition was adjusted for mtDNA linearization. Following 2 h of incubation at 37 °C, the restriction enzyme was inhibited using 65 °C for 20 min. Subsequently, 20 units of RNase I were added, and the sample was further incubated for 1 h at 37 °C. Finally, 0.8 units of ProK were added, and the sample was incubated for an additional 1 h at 37 °C.

### Preparation of mtDNA and syDNA Samples for Electro-Optical Nanopore Analysis

We used mtDNA extracted from isolated mitochondria of human placenta, alongside synthetic NoLimits DNA of 17 kb (syDNA). 1 nM of each of these two samples was subjected to primary amine-specific labeling chemistry using Atto565 NHS ester (Atto-tec) at 500 nM in a buffer containing 10 mM sodium phosphate and 150 mM NaCl (pH 8.4) for 1 h at RT. The unconjugated dye was removed using dialysis with mini-dialysis tubes of 20 kDa cutoff against 20 mM Tris-acetate buffer (pH 7.9) compatible with subsequent processing steps. Then, the samples were adjusted to ExoV reaction conditions using NEBuffer 4 and ATP. The mtDNA sample was treated with ExoV for 1 h at 37 °C, whereas the syDNA sample was subjected to an identical treatment but using a preinhibited ExoV instead. After ExoV inhibition, both samples were adjusted with BSA and the rCutSmart buffer. Subsequently, both were incubated with AflII for 2 h at 37 °C, resulting in mtDNA linearization, whereas the syDNA, which lacks an AflII recognition site, was not cut by this restriction enzyme (confirmed experimentally by gel electrophoresis—data not shown). Linearization of the mtDNA sample and intactness of the syDNA sample, following this complete procedure, were confirmed by gel electrophoresis poststained with SybrGold (data not shown). Finally, these two samples were subjected to careful analysis using our custom-built Optipore system.

### Nanopore Fabrication and Device Assembly

Nanopore chips were fabricated on 4-inch silicon wafers, which were coated with a 500 nm layer of silicon dioxide (SiO_2_) and a 50 nm layer of low-stress amorphous silicon nitride (SiN_*x*_). Reactive ion etching (RIE) was used to locally thin the SiN_*x*_ to 8–10 nm, creating approximately 2 μm diameter wells. Following this, wet etching with buffered hydrofluoric acid (HF) removed the underlying SiO_2_. The etched regions of SiN_*x*_ and SiO_2_ served as a hard mask for anisotropic silicon etching in potassium hydroxide (33% KOH).^29^ The devices were subsequently cleaned using a 2:1 solution of sulfuric acid (H_2_SO4) and hydrogen peroxide (H_2_O_2_), and then integrated into a Teflon flow cell. A buffer solution (1 M KCl, 40 mM Tris-HCl, 1 mM EDTA, pH 7.5), filtered with a 0.02 μm syringe filter, was used to immerse the devices. Two Ag/AgCl wire electrodes were connected to an Axon Axopatch 200B amplifier for monitoring. Nanopores were created in the thinned SiN_*x*_ regions using controlled dielectric breakdown (CBD), as previously described.^55,56^ The CBD process was managed with a custom-built voltage/current amplifier and LabVIEW software. Nanopore diameters were controlled within the range of 5–6 nm, which was found to be optimal for detecting both long mtDNA molecules and smaller fragments.

### Data Acquisition and Analysis

For data acquisition, an open-pore current was established by applying a bias voltage of 100–200 mV across the nanopore’s *cis* chamber. Once stable ionic flow with minimal noise was achieved, the device was ready for use. The translocation events were recorded by using the Axon 200B amplifier, filtered through a 100 kHz low-pass filter, and analyzed with custom LabVIEW software. The program identified each event and extracted key parameters, such as the fractional blockage current (*I*_B_), dwell time (*t*_D_), and the standard deviation of the blockage current (*i*_rms_). The data were then processed using Igor Pro 8 for statistical analysis and visualization, including histograms, scatter plots, and curve fitting.

### Electro-Optical Acquisition System

A custom-designed wide-field microscope was constructed for imaging and simultaneously monitoring the fluorescent DNA translocations through the nanopore. In this microscope, a 532-nm (Cobolt Samba) laser was coupled to a single-mode fiber and further collimated and focused on the back aperture of an objective lens. A 60× oil immersion objective lens (Olympus PlanApo, 1.5 NA) was used to create a laser spot of ∼6 μm diameter illumination area. The fluorescence signals were filtered from the illumination laser using multiband dichroic mirrors (ZT405/488/532/640rpc Chroma) and filters (Chroma, ZET532/NF). Images were acquired on an Andor EMCCD (Andor iXon+^EM^ DU-860) at 30 × 30 cropped sensor mode with 1 ms exposure and 1 × 1-pixel binning.

### Data Acquisition and Analysis for Electro-Optical Measurement

For labeling 1 nM syDNA with POPO-3 dye (Thermo Fisher), we used the recommended protocol (Dimeric Cyanine Nucleic Acid Stains User Guide, Doc. Part No. MP03600, Pub. No. MAN0001843) to achieve a 1:10 dye-to-base pair ratio. For all three DNA samples (syDNA + Popo-3, syDNA + Atto-565 NHS ester, and mtDNA + Atto-565 NHS ester), the final nanopore experiment was carried out by diluting the DNA samples to a final concentration of 30 pM in the *cis* chamber. To apply the transmembrane voltages, an Axon 200B patch clamp was used. A 5 mW laser power was sufficient to acquire fluorescence signals from highly labeled DNA samples without an optical background. A computer-controlled custom LabView software was used to simultaneously control the voltage and record the current after a 10 kHz bandpass filter and translocation-driven optical signals on an EMCCD. Custom LabView and MATLAB programs were written to analyze the translocation-driven optical measurements, and further graphics were generated in the Igor Pro software.

### Machine Learning Workflow

#### Creation of the Training Set

The machine learning model for event classification was developed using MATLAB’s Classification Learner toolbox (R2024a). A multiclass one-vs-one support vector machine (SVM) algorithm with a fine Gaussian kernel (kernel scale = 2) was employed. Our classification process involved two distinct steps: training the SVM model and subsequently using the trained model to classify low-abundance clinical samples. During the training phase, we utilized pure samples that provided many translocation events. Key event features (*I*_B_, *t*_D_, and *i*_rms_) were extracted and employed in the training.

Current blockage values were normalized relative to the open-pore current, and logarithmic values of dwell times were used to reduce the data range variations that could introduce errors in the SVM model. Different multiclass SVM models were trained on data sets derived from pure samples of synthetic DNA (syDNA), mtDNA, mtDNA fragments, and pcDNA3.1. Initially, a three-class model was trained to demonstrate that the SVM could identify mtDNA with high accuracy even in the presence of syDNA of the same length and a background of fragmented DNA. For the final analysis, the model was retrained for two-class classification, as clinical samples contained only mtDNA and gDNA fragments, with no syDNA.

#### SVM Model Validation

To validate the model’s classification performance, we used metrics such as accuracy, confusion matrix, true positive rate, and 5-fold cross-validation. Data randomization ensured the robustness of the model and prevented overfitting. ROC plots with AUC values exceeding 0.9 for all classes confirmed the reliability of our trained models. Additionally, we verified the model’s performance using ratiometric analysis of plasmid mixtures. The SVM-derived ratios closely matched experimental values within error limits, further demonstrating the model’s reliability. This ensured that the SVM model is robust and capable of accurately classifying mtDNA events across different experimental conditions. This trained model was used in the final analysis of endogenous samples, in which it automatically classified events based on the learned data, assigning them to the appropriate group.

## Supporting Information Available

Biochemical assay validation using mtDNA or plasmids, biochemical assay modification and validation using total DNA samples from cells, nanopore and qPCR measurements of mtDNA to gDNA copy number ratios (PDF)
Nanopore translocation recordings for the mtDNA-enriched total DNA sample (MP4)
