# Supplementary material for: Amplification-Free Quantification of Endogenous Mitochondrial DNA Copy Number Using Solid-State Nanopores
Source: ACS Nano. 2025 Mar 13;19(11):11390–402. doi: 10.1021/acsnano.5c00732 (PMC11948453; doi:10.1021/acsnano.5c00732)
Supplement: Supplementary file 1 — nn5c00732_si_001.pdf [file nn5c00732_si_001.pdf]

Supporting Information

Amplification-free quantification of endogenous mitochondrial DNA copy number using solid-state nanopores

Sohini Pal[1,#], Diana Huttner[1,#], Navneet C. Verma[1], Talya Nemirovsky[1], Oren Ziv[2], Noa Sher[2], Natalie Yivgi-Ohana[2] and Amit Meller[1,3,*]

1. *Faculty of Biomedical Engineering, Technion -IIT, Haifa, 3200003, Israel*
2. *Minovia Therapeutics Ltd., Tirat Carmel, 3902603, Israel*
3. *Russell Berrie Nanotechnology Institute, Technion -IIT, Haifa, 3200003, Israel*

*Corresponding author, E-mail: ameller@technion.ac.il
# equally contributing authors

List of the Supporting Information Figures, Tables and Videos:

Figure S1 – Preparation of linearized mtDNA samples for nanopore sensing validation using PvuII as a restriction enzyme.

Figure S2 – Comparison of enzymatic restriction efficiency under optimal buffer conditions or using ExoV buffer conditions.

Figure S3 – Protocol validation using plasmids as a model for circular mtDNA.

Figure S4 – Sample preparation does not cause the mtDNA-specific ion-current fluctuations.

Figure S5 – Gel validation of ATP regeneration system requirement for efficient fragmentation of gDNA by ExoV.

Figure S6 – Gel analysis of samples of total DNA from HCT116 spiked with different mtDNA ratios.

Figure S7 – Analysis of total DNA from CD34$^+$ spiked with different mtDNA ratios and analysis of endogenic mtDNA in HCT116 or CD34$^+$ cells.

Table S1 – Summary of qPCR analysis of total DNA extracted from PBMCs sample.

Figure S8 – Nanopore analysis of the PBMCs sample used also in the qPCR calibration.

Figure S9 – Nanopore analysis of the Rho Zero cell line sample.

Table S2 - Summary of the data used for main text Figure 4.

Supporting video SI Movie 1 – Continuous recording of a representative nanopore experiment.

1. Biochemical assay validation using mtDNA or plasmids

Protocol validation using mtDNA from isolated mitochondria

mtDNA was extracted from isolated mitochondria of human placenta, subjected to our biochemical assay (explained in Figure 3a), separated on a 0.6% agarose gel in 1xTAE buffer, post-stained with SybrGold and imaged using the Pharos gel scanner (Figure S1). Before the biochemical assay, mtDNA runs as a supercoiled circular molecule (lane 1) with varying amounts of residual linear gDNA (compare also with Figure 1a, lane 1). Short ExoV treatment was used in this case to digest any residual contaminating linear gDNA, resulting in intact circular supercoiled mtDNA (lane 2). Following ExoV inhibition and buffer adjustment, mtDNA was linearized using PvuII, which similar to AflII, targets a highly consensus region in the mtDNA. As expected, PvuII-linearized mtDNA runs as a single band of ~17,000 bp (lane 3), similarly to AflII-linearized mtDNA (compare with Figure 1a, lane 2). PvuII-linearized mtDNA was further subjected to enzymatic fragmentation, using AluI and HaeIII, resulting in shorter fragments, which were undetected by SybrGold staining (lane 4). This result is consistent with fragmented mtDNA, shown in Figure 1a, lane 3. PvuII-linearized mtDNA or fragmented mtDNA samples, were analyzed in ssNPs separately, showing comparable results to Figure 1b and 1c, middle panels or bottom panels, respectively (data not shown).

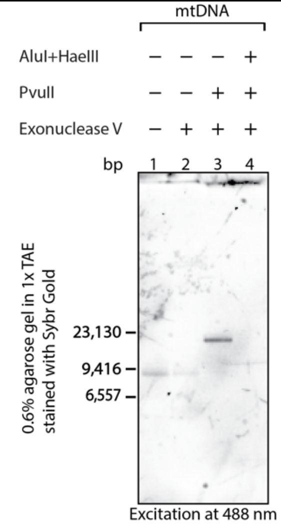

Figure S1 – Preparation of linearized mtDNA samples for nanopore sensing validation using PvuII as a restriction enzyme. mtDNA, extracted from isolated mitochondria of human placenta, was subjected to ExoV treatment for 1 h at 37°C. Following inhibition at 70°C for 30 min. and subsequent buffer adjustment with BSA, mtDNA was incubated with PvuII for 2 h at 37°C in the presence or absence of AluI and HaeIII. The enzymes were inhibited at 80°C for 20 minutes. Finally, ProK was added to the samples for further incubation for 1 h at 37°C. The samples were analyzed on 0.6% agarose in 1xTAE. After separation, the gel was stained in 1x Sybr Gold for 1 h at RT with gentle shaking. Image was acquired with the Pharos imager using excitation at 488 nm.

Protocol validation using purified plasmids

Plasmids (pcDNA3.1 (+) and pUC18) were each transfected into XL10-gold ultracompetent cells, and grown on LB-agar plates containing ampicillin, chloramphenicol and tetracycline. Isolated colonies were inoculated in LB with ampicillin and grown at 37°C at 300 rpm for overnight. Plasmids were extracted

using a kit (Wizard SV minipreps kit, Promega), validated by restriction enzyme digest (data not shown) and used for protocol validation as model circular molecules.

To establish buffer compatibility and efficient linearization protocol, the plasmid cleavage by each restriction enzyme was compared under optimal conditions (using NEB rCutSmart buffer) to plasmid linearization using ExoV buffer conditions (NEBuffer 4 and ATP), supplemented with bovine serum albumin (BSA). As shown in Figure S2, incubation of ExoV with the plasmid did *not* alter its circular supercoiled structure. Furthermore, cleavage of the plasmid with either one of the three enzymes (AflII, PvuII or BamHI) following ExoV inhibition and buffer adjustments showed comparable efficiency to plasmid cleavage performed under optimal buffer conditions in the absence of ExoV. Furthermore, the results suggest that ExoV inhibition was efficient, as the intensity of the BamHI- or AflII-linearized bands was comparable in the presence or absence of ExoV. Albeit, to confirm that ExoV was indeed active, in parallel to pcDNA3.1 plasmid treatment, we included a linear dsDNA molecule (syDNA 5000 bp NoLimits DNA) as a positive control (see Figure S3A, before (lane 1) or after (lane 2) ExoV treatment). This result showed that the applied short exonuclease treatment was sufficient to digest linear dsDNA, of a similar size to of the plasmid, below the SybrSafe staining detection limit (lane 2), while preserving the circular integrity of the pcDNA3.1 plasmid (lane 3). Efficient linearization (BamHI or AflII, lane 4 or 5, respectively) or cleavage at three sites (PvuII, lane 6) was observed using the pcDNA3.1 plasmid, and, as expected, further incubation with ProK to digest the enzymes did not affect the results on the gel (compare Figure S3A with Figure S2) or the results in ssNPs analysis (see Figure 3b, right panels).

To confirm that the biochemical assay is suitable for sample preparation for ssNPs experiments we subjected pcDNA3.1 (see Figure 3b, middle panel) or pUC18 (see Figure S3B) to the complete process described in Figure 3a, in the presence or absence of the restriction enzyme. The samples were separated on a 0.6% agarose gel containing 1xSybrSafe in 1xTAE buffer and imaged using the GelDoc apparatus. Both linearized pcDNA3.1 or pUC18 are seen as a single band in the gel, which corresponds to their expected size, 5428 bp or 2686 bp, respectively. Linearized pcDNA3.1 or pUC18 samples were subjected to ssNPs analysis, Figure 3b (right panels) or Figure S3C, respectively. Nanopore translocations in each case show a single population, suggesting that their structural integrity is preserved in the biochemical assay.

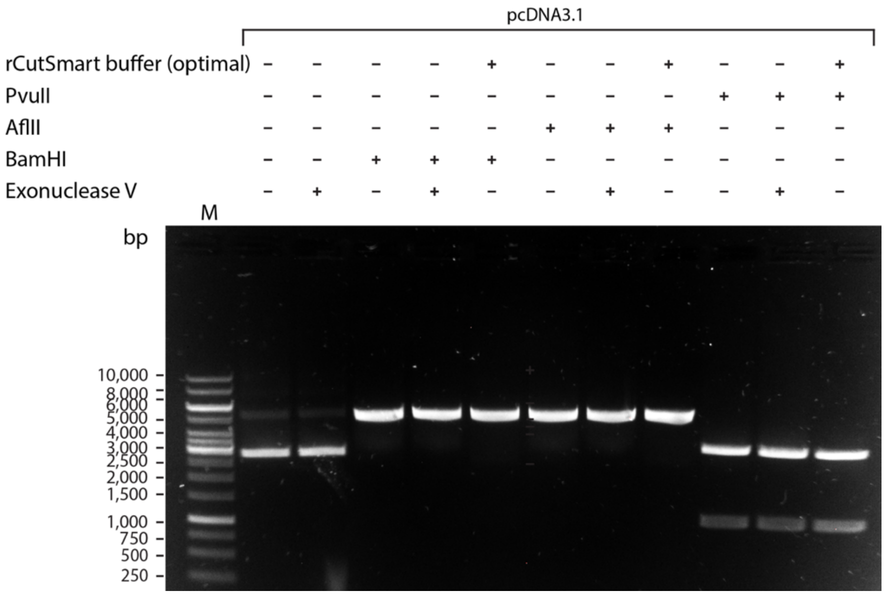

Figure S2 – Comparison of enzymatic restriction efficiency under optimal buffer conditions or using ExoV buffer conditions. Plasmid pcDNA3.1 was incubated with a restriction enzyme (BamHI, AflII or PvuII) either following ExoV treatment and inhibition (in the presence of 1xNEBuffer 4+ATP, then supplemented with BSA prior to restriction enzyme addition) or directly using rCutSmart buffer (optimal conditions) or using instead NEBuffer 4 supplemented with ATP and BSA (but in the absence of ExoV). Samples were separated on 0.8% agarose gel in 1xTAE buffer supplemented with 1x SybrSafe and imaged using the GelDoc apparatus. GeneRuler 1kb DNA ladder was used as a size reference marker (lane M).

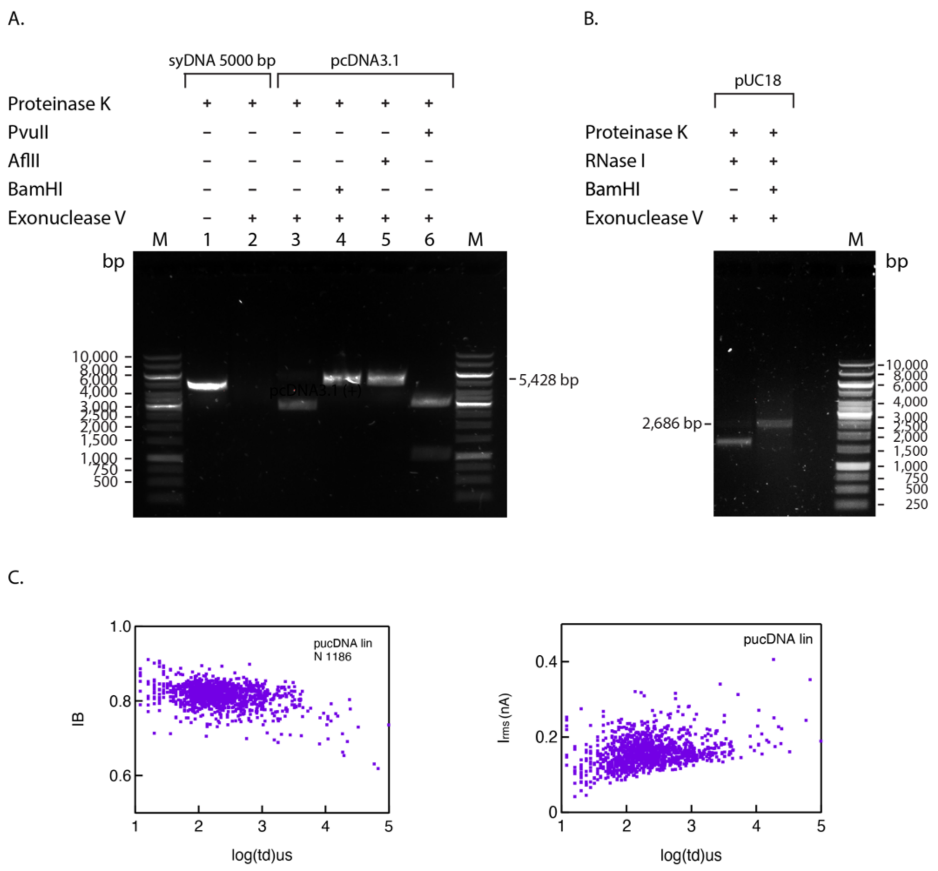

Figure S3 – Protocol validation using plasmids as a model for circular mtDNA. A. NoLimits DNA (depicted as syDNA 5000 bp) was incubated for 1 h at 37ºC with (lane 2) or without (lane 1) ExoV, followed by enzyme inhibition at 70ºC for 30 minutes and enzyme digest with ProK for an additional 1 h at 37ºC. Plasmid pcDNA3.1 was subjected to the same ExoV treatment, followed by inhibition and subsequent cleavage by a restriction enzyme. Description of lanes: no enzyme (lane 3), BamHI (lane 4), AflII (lane 5) or PvuII (lane 6). Treatment was finalized by incubation with ProK for one hour at 37ºC. B. pUC18 plasmid was subjected to ExoV treatment for 1 h at 37ºC. Following inhibition, the plasmid was incubated with or without BamHI for 2 h at 37ºC, as depicted in the image. The treatment was finalized by incubation with RNaseI for 1 h at 37ºC, followed ProK incubation for additional 1 h at 37ºC. The samples of A. and B. were separated on 0.8% agarose gels in 1xTAE buffer supplemented with 1x SybrSafe and the image was acquired using the GelDoc apparatus. C. Corresponding scatter plots for linearized pUC18 (from panel B) translocations with I_B, log(t_D), and std show a single population (N = 1186), suggesting that their structural integrity is preserved in the biochemical assay.

Sample preparation does not cause the mtDNA-specific ion-current fluctuations

To exclude the possibility that the sample preparation procedure may cause the ion-current fluctuations observed for the mtDNA sample in the ssNPs experiments, we analyzed in parallel mtDNA extracted from isolated mitochondria of human placenta and syDNA (NoLimits DNA 17 kbp fragment). The mtDNA sample was treated with ExoV for 1 h at 37ºC, whereas the syDNA sample was subjected to an identical treatment but using a pre-inhibited ExoV instead. ExoV was then inhibited for 30 minutes at 70ºC. Subsequently, either sample was incubated with AflII for 2 h at 37ºC, resulting in mtDNA linearization,

whereas the syDNA was not cut by this restriction enzyme. Subsequently, both sample was subjected to ProK treatment for 1 h at 37ºC. These samples were separated on a 0.6% agarose gel in 1xTAE buffer, post-stained with SybrGold and imaged using the Pharos gel scanner (Figure S4A) prior to ssNP experiments and SVM analysis. ssNP translocations for syDNA showed an average i_{rms} value of **0.19 ± 0.05 nA** with only 3.8% of the syDNA events showing high i_{rms} of 1.1 ± 0.1 nA, consistent with Figure 2d in the manuscript, showing that the biochemical treatment is not the source of the near 6-fold increase in RMS. Representative translocation events of the syDNA control sample are shown in Figure S4B.

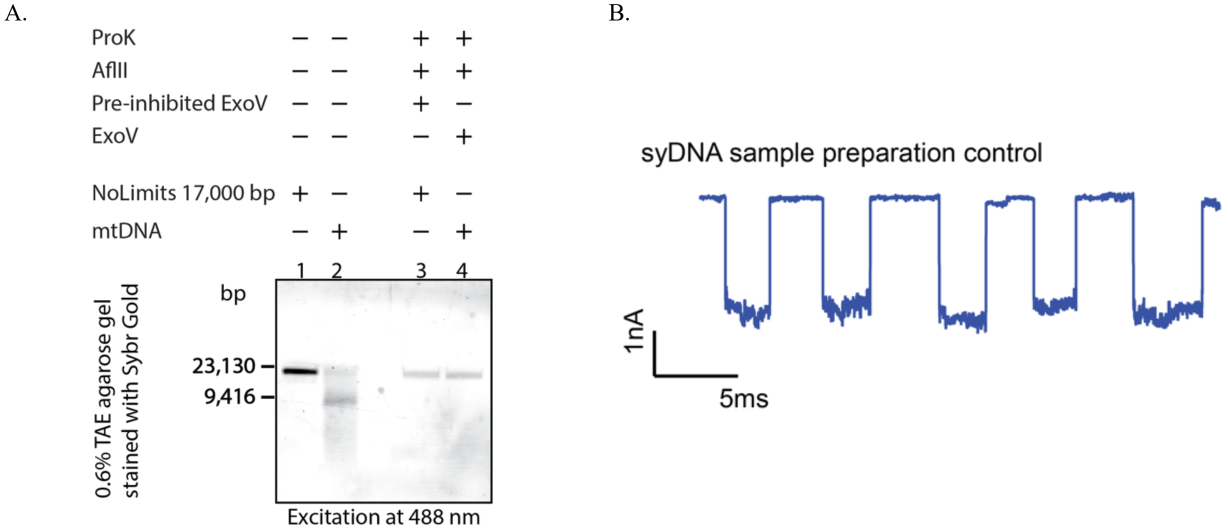

Figure S4 – Sample preparation does not cause the mtDNA-specific ion-current fluctuations. A. Linear NoLimits DNA of 17,000 bp (also depicted as syDNA) (lane 1) or supercoiled circular mtDNA extracted from isolated mitochondria of human placenta (lane 2) before treatment. NoLimits DNA of 17,000 bp (lane 3) or mtDNA (lane 4) samples following the complete sample preparation protocol. B. Representative ion-current translocations of this sample preparation control syDNA are consistent with those shown in Figure 1c (upper panel).

2. Biochemical assay modification and validation using total DNA samples from cells

Total DNA samples mostly contain linear genomic DNA (gDNA) and only a small fraction of circular mtDNA (by mass). In order to reduce gDNA background and enrich for mtDNA translocations in a controllable manner, we modified the sample preparation protocol, specifically at the ExoV linear dsDNA fragmentation step, as described below. HCT116 cells were purchased from American Type Culture Collection (ATCC), grown in DMEM media supplemented with 10% foetal calf serum and antibiotics at 37ºC in flasks in a humidified incubator at 37 °C with 5% CO_2. The cells were harvested at 80% confluency for subsequent total DNA extraction, using Invitrogen Purelink genomic DNA mini kit (Thermo Fisher Scientific). Total DNA was eluted with 10 mM Tris-HCl buffer (pH 8) . Total DNA concentration was quantified using a nanodrop measuring the absorbance at 260 nm. The purity of the preparation was validated by measuring the ratio absorbance of 260 nm / 280 nm.

Preliminary time course experiments using 20 units of ExoV with 1 mM ATP analyzed in Tape Station (Agilent 4200) suggested that ExoV loses its activity after a maximum of four hours at 37ºC (data not shown). We postulated that the essential ATP co-factor is exhausted by that time. Since according to New England Biolabs product datasheet, excess of ATP may result in ExoV inhibition, we instead used an ATP regeneration system to allow for prolonged, extensive and efficient ExoV activity. In Figure S5, equal amounts of total DNA were subjected to ExoV treatment in the presence and absence of ATP regeneration system for O/N at 37ºC. Tape Station analysis demonstrates that in the presence of the ATP regeneration system, the typical peak of gDNA (~50 kb) disappears after an O/N incubation, whereas in the absence of ATP regeneration, this peak persisted. Nevertheless, short gDNA fragments are still present in the sample, as observed in the Tape Station analysis, even after an overnight incubation (Figure S5, lane D1). As described in the Methods section, we opt for applying four overnights with two additions of fresh ExoV, ATP and the ATP regeneration system in order to reach a steady state of fragmented gDNA.

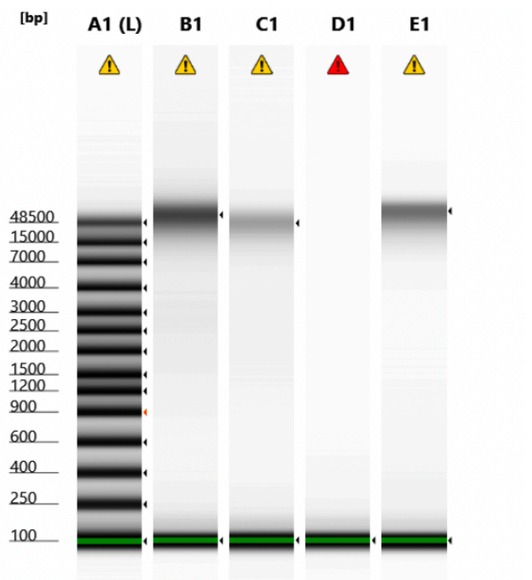

Figure S5 – Gel validation of ATP regeneration system requirement for efficient fragmentation of gDNA by ExoV. Equal amounts of total DNA extracted from HCT116 were subjected to ExoV treatment in the presence and absence of an ATP regeneration system for O/N at 37ºC and analyzed by Tape Station using the genomic DNA kit. Lane A1, DNA ladder, lane B1, gDNA sample before adding ExoV, lane C1, gDNA sample after adding ExoV in the presence of ATP regeneration system (0 h incubation), lane D1, gDNA sample after adding ExoV in the presence of ATP regeneration system (O/N incubation at 37ºC), and lane E1, gDNA sample after adding ExoV w/o ATP regeneration system (O/N incubation at 37ºC). Although O/N incubation with ExoV and ATP regeneration system results in the disappearance of the major heavy band (lane D1), short DNA fragments persist in the sample.

Extensive ExoV treatment does not alter mtDNA copy number

ExoV has a bi-directional exonuclease activity, which requires dsDNA or ssDNA ends for its activity, thereby it theoretically cannot digest circular DNA molecules, such as mtDNA. However, in order to demonstrate that the four overnight ExoV treatment we used on total DNA preparations did not result in mtDNA copy number alteration, we performed the following experiment (Figure S7A). We applied the biochemical assay on 200 ng of total DNA CD34^{+} cells from a healthy donor, spiked with 20 ng of mtDNA, in parallel to a sample containing only 20 ng of mtDNA. Following the complete assay, these samples were separated on 0.6% agarose gel in 1xTAE buffer, post-stained with SybrGold. Then, the gel image was

acquired as before. Importantly, the linearized mtDNA band intensity obtained in these two samples following the biochemical assay is similar (Figure S7A).

To further show that our biochemical assay preserves mtDNA copy number, we performed three separated experiments. Two of which total DNA from HCT116 cells (Figure S6A and S6B), and one with total DNA from $CD34^+$ cells of a healthy donor (Figure S7A), were spiked with different amounts of mtDNA from isolated mitochondria. Following the biochemical assay, the samples were separated on 0.6% agarose gel in 1xTAE buffer, post-stained with SybrGold. Then, the gel images were acquired using the Pharos imager at 488 nm excitation. As observed in all three images, the linearized mtDNA band stains in good correspondence to the initial amount of spiked mtDNA. Noticeably, the ssNPs experiments carried out with these samples and their subsequent SVM analysis are presented in Figure 4c (selected mtDNA-spiked HCT116) or in Figure S7B (mtDNA-spiked $CD34^+$). The summarized results of all spiked-mtDNA samples are shown in Table S2. Moreover, main text Figure 4d (mtDNA-spiked HCT116 samples) show a good fit to our model presented below (Supporting Information point 3). Importantly, the predicted endogenous mtDNA:gDNA ratio in the HCT116 sample is consistent with our fit, strongly suggesting that ExoV extensive treatment preserves mtDNA for proper quantification.

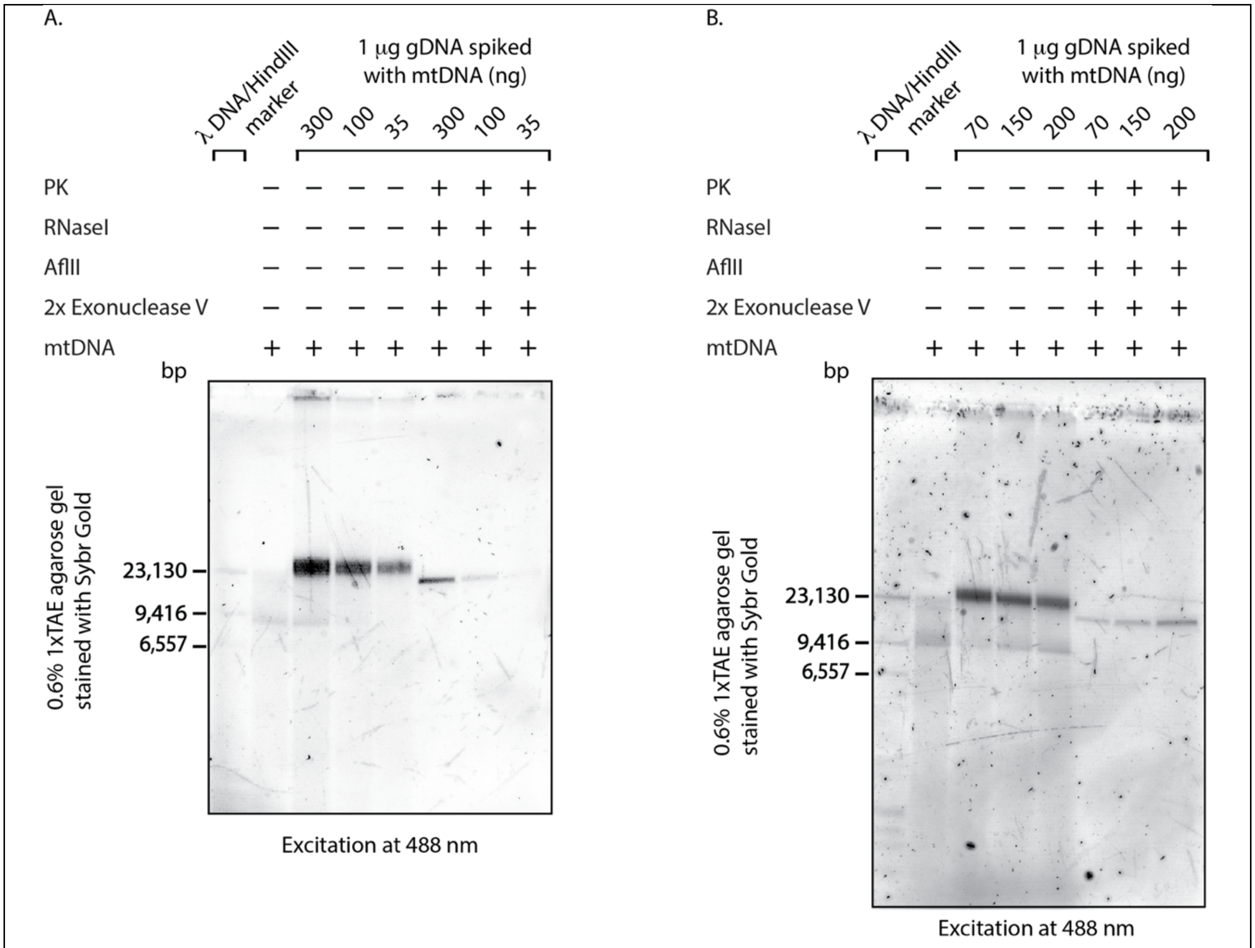

Figure S6 – Gel analysis of samples of total DNA from HCT116 spiked with different mtDNA ratios. 1 µg of total DNA, extracted from HCT116 cancer cells, was spiked with mtDNA, from isolated mitochondria using 35, 100 and 300 ng (A) or 70, 150 and 200 ng (B). The samples were subjected to ExoV treatment for 4 overnights at 37ºC. Following inhibition at 70ºC for 30 min. and subsequent buffer adjustment with BSA, AflII was added for 2 h at 37ºC. Subsequently, the enzyme was inhibited at 65ºC for 20 minutes. Then, RNaseI was added for 1 h at 37ºC. Finally, ProK was added to the samples for further incubation for 1 h at 37ºC. The samples were analyzed on 0.6% agarose in 1xTAE, stained 1x Sybr Gold for 1 h at RT. Image was acquired with the Pharos gel scanner (BioRad).

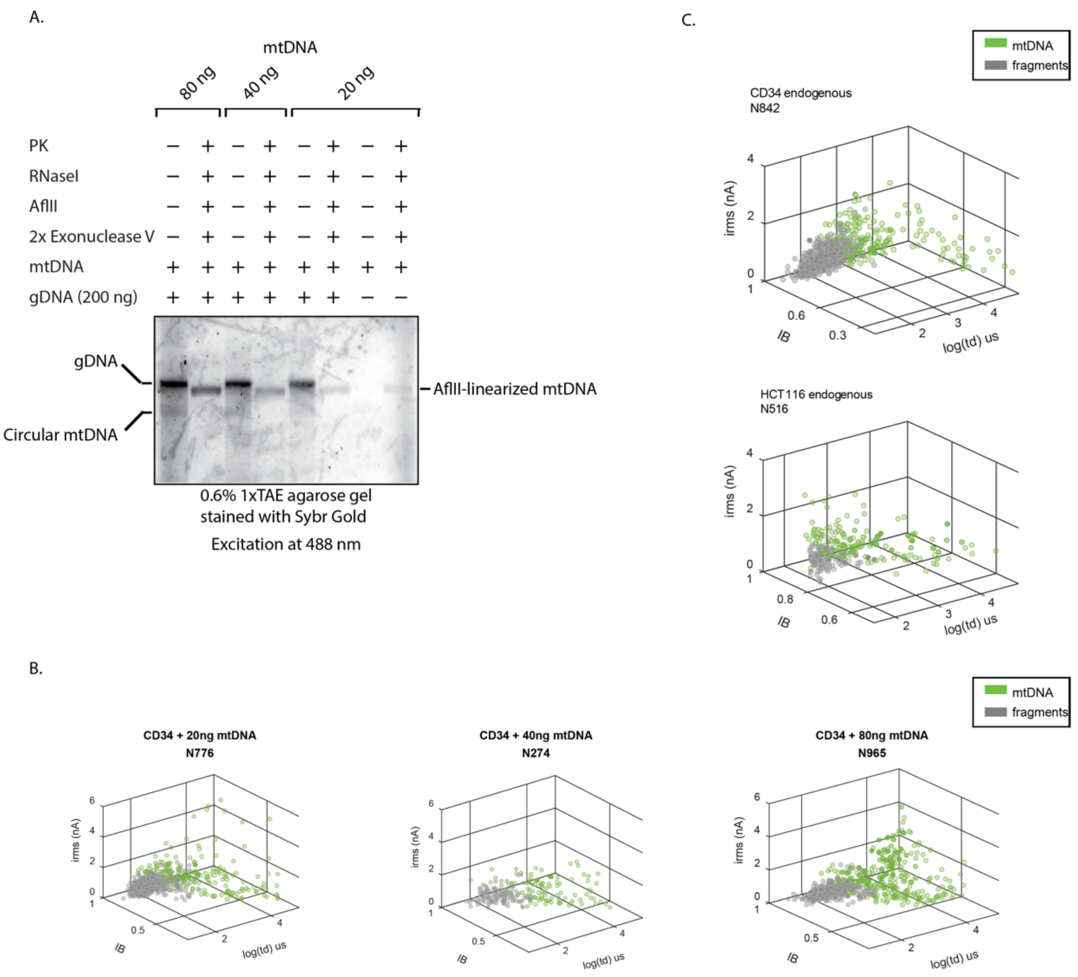

Figure S7 – Analysis of total DNA from $CD34^+$ spiked with different mtDNA ratios and analysis of endogenic mtDNA in HCT116 or $CD34^+$ cells. (A) 200 ng of total DNA, extracted from $CD34^+$ cells, spiked with mtDNA, from isolated mitochondria using 20, 40 and 80 ng. The samples were subjected to ExoV treatment for 4 overnights at 37ºC. Following inhibition at 70ºC for 30 min. and subsequent buffer adjustment with BSA, AflII was added for 2 h at 37ºC. Subsequently, the enzyme was inhibited at 65ºC for 20 minutes. Then, RNaseI was added for 1 h at 37ºC. Finally, ProK was added to the samples for further incubation for 1 h at 37ºC. The samples were analyzed on 0.6% agarose gel in 1xTAE, stained in 1x Sybr Gold for 1 h at RT. (B) 3D scatter plots representing the classification of spike-in mtDNA in $CD34^+$ cells at varying amounts 20 ng (left), 40 ng (middle), and 80 ng (right). Each dataset shows progressively increasing densities of mtDNA (green dots), indicating successful classification across different spike-in concentrations. (C) 3D scatter plots showing endogenous mtDNA detection in $CD34^+$ cells (upper, N=842) and HCT116 cells (lower, N=516). Green dots represent detected mtDNA events, with a relatively sparse distribution compared to the spike-in datasets, reflecting lower endogenous levels.

3. **Nanopore and qPCR measurements of mtDNA to gDNA copy number ratios**

Following Rooney et al., 2015[1]*, we estimate the* mtDNA to genomic DNA *copy number ratio* measure by qPCR *by:*

$$R_{PCR} = \frac{N_{mt}}{N_g} \approx 2 \cdot 2^{\Delta C_t}$$

Where ΔC_t is the difference in the qPCR C_t values between the genomic (or nuclear) gene and the mtDNA. We analyzed the same PBMC sample by qPCR and the nanopore method. Total DNA was extracted from PBMCs of a healthy donor[#] using a commercial kit (Qiagen) according to the manufacturer instructions and quantified using Qubit, qPCR results (performed in triplicate reactions) are summarized in Table S1 and nanopore results in Figure S8. For our PBMC sample we get $R_{PCR} = 59.5 \pm 2.5$.

To calculate the fraction of mtDNA to the total DNA content in sample we use:

$$R = \frac{N_{mt}}{N_{mt}+N_g} = \frac{1}{1+\frac{1}{R_{PCR}}} \qquad \text{(Eq. 1)}$$

Sample	C_t average mtDNA target (FAM)	C_t average nucDNA target (VIC)	ΔCt			2 x $2^{(\Delta Ct)}$			
			aliquot1	aliquot2	aliquot3	aliquot1	aliquot2	aliquot3	average
Control PBMCs sample	21.24	26.13	4.82	4.94	4.91	56.6	61.4	60.4	59.5 ± 2.5

Table S1 Summary of qPCR analysis of total DNA extracted from PBMCs sample.

To convert Eq. 1 to mass/mass ratio we scale it by the length (base pairs) ratio of gDNA to mtDNA molecules, giving percentage ratio: $R'(\%) \sim 0.034$. Similarly, for the Nanopore case the fraction of mtDNA to the total DNA content in the nanopore sample is:

$$R_{NP} = \frac{N_{mt}}{N_{mt}+N_g^*} = \frac{1}{1+\frac{N_g^*}{N_{mt}}} \qquad \text{(Eq. 2)}$$

where N_g^* is the adjusted copy number of the genomic DNAs after the biochemical process. When analyzing the same sample, we can relate the nanopore and qPCR ratios by:

$$R_{NP} = \frac{1}{1+\beta/R_{PCR}} \qquad \text{(Eq. 3)}$$

[#] Peripheral blood sample was obtained from a healthy donor in the Hadassah hospital, Jerusalem, Israel, for the LiquidBx consortium according to the regulations of the Clinical Research and Ethics Committee and the Helsinki Declaration of the World Medical Association (HMO 198-14).

where the constant β can be determined experimentally. Using results from Figure S8 ($R_{NP} = 0.286 \pm 0.014$) we obtain: $\beta = 148.9 \pm 7.4$.

We can therefore express the mtDNA to total DNA fraction (Eq. 1) in terms of the measured R_{NP} as:

$$R = \frac{\beta R_{NP}}{1 + (\beta - 1)R_{NP}} \approx \frac{\beta R_{NP}}{1 + \beta R_{NP}}$$

Or expressed in mass/mass percentage as:

$$R'(\%) = \frac{100}{1+\left(\frac{1-R_{NP}}{\beta R_{NP}}\right)x} \qquad \text{(Eq. 4)}$$

where x is the ratio of the gDNA to mtDNA base pairs.

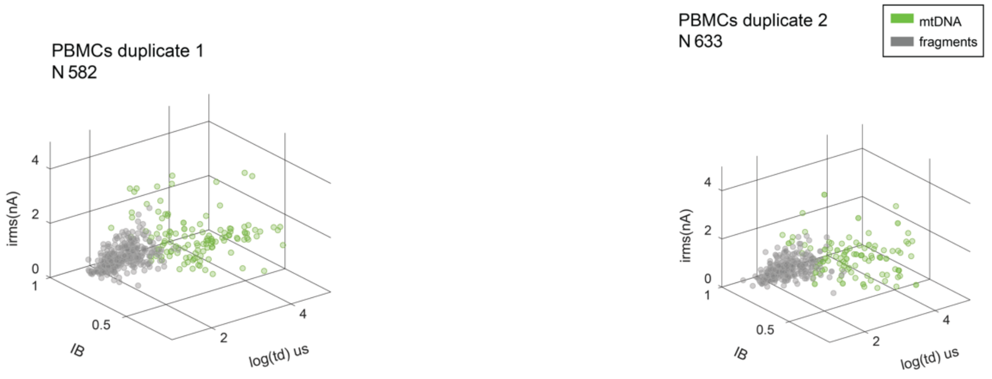

Sample	Total (#)	mtDNA (SVM)	R_{NP}
**PBMCs duplicate 1**	582	168 ± 4	**0.288 ± 0.008**
**PBMCs duplicate 2**	633	633 ± 5	**0.283 ± 0.007**

Figure S8 – Nanopore analysis of the PBMCs sample used also in the qPCR calibration.
Total DNA was extracted from PBMCs of a healthy donor and analyzed in parallel by quantitative real-time PCR (qPCR) and by our biochemical process followed by ssNPs analysis. The ratio of mtDNA translocations, as identified using the SVM, from the total number of translocations is presented (upper panel, mtDNA in green and gDNA fragments in grey). As shown in the summarized table (lower panel), the measured mtDNA to gDNA ratio was almost identical for the two duplicate samples, within the error margins.

Nanopore measurement of mtDNA-depleted cells lines Rho Zero

The cell line A549p0#1a (catalog #ESA113, Kerafast) underwent a process for mtDNA depletion,[2] hence it is a useful sample to challenge the sensitivity and accuracy of our method. Total DNA was extracted from

this cell line using a commercial kit (Qiagen) according to the manufacturer instructions and quantified using Qubit. Depletion of mtDNA was confirmed by quantitative PCR (qPCR) analysis with undetermined (below detection limit) C_t values for the mtDNA-encoded target (data not shown). Subsequently, we used this sample in our biochemical process followed by ssNP experiments and SVM analysis (Figure S9). Accordingly, we obtain R_{NP}= 0.068±0.004, or $R'(\%)$ = 0.006%.

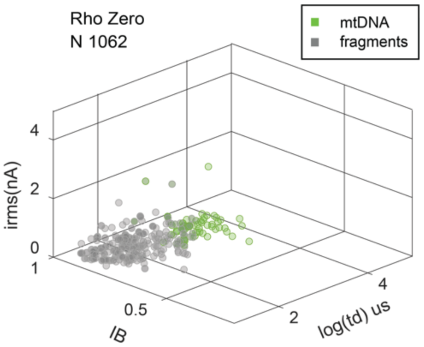

Sample	Total (#)	mtDNA (SVM)	R_{NP}	R' (%)
**Rho Zero**	1062	73 ± 4	0.068 ± 0.004	**0.006**
**HCT116 spiked with 300ng mtDNA**	1705	1199 ± 32	0.703 ± 0.002	**0.20**

Figure S9 – Nanopore analysis of the Rho Zero mtDNA depleted cell line.
Total DNA was extracted from Rho Zero cell line and analyzed by our biochemical process followed by ssNPs analysis. The ratio of mtDNA translocations, as identified using the SVM, from the total number of translocations is presented in the scatter plot (mtDNA in green and gDNA fragments in grey) and in the table. The results for the HCT116 total DNA sample spiked with 300 ng mtDNA are shown in parallel (taken from Table S2) to demonstrate the dynamic range of our method.

Sample	Total (#)	mtDNA (SVM)	R_{NP}	R' (%)
**HCT116**				
Endogenous mtDNA	516	140 ± 4	0.271 ± 0.008	**0.031**
35 ng mtDNA	1118	376 ± 10	0.336 ± 0.009	**0.0427**
70 ng mtDNA	1218	507 ± 14	0.416 ± 0.001	**0.060**
100 ng mtDNA	1137	592 ± 16	0.521 ± 0.001	**0.092**
150 ng mtDNA	667	355 ± 10	0.532 ± 0.002	**0.096**
200 ng mtDNA	797	483 ± 13	0.606 ± 0.002	**0.13**
300 ng mtDNA	1705	1199 ± 32	0.703 ± 0.002	**0.20**
**CD34**				
Endogenous mtDNA	842	202 ± 6	0.240 ± 0.007	**0.0267**
20 ng mtDNA	776	276 ± 7	0.356 ± 0.009	**0.047**
40n g mtDNA	274	148 ± 4	0.540 ± 0.001	**0.99**
80 ng mtDNA	965	583 ± 15	0.604 ± 0.002	**0.13**

Table S2 Summary of the data used for main text Figure 4, showing total event counts, mtDNA counts (as determined by SVM classification), and the calculated ratio (mtDNA events to total events) for each sample type.

Caption for the supported video:

Supporting video SI Movie 1 – Continuous recording of a representative nanopore experiment.

The video shows stable open pore current before the sample is added. Once HCT116 total DNA sample spiked with mtDNA is added to the pore, representative translocation events of mtDNA and short gDNA fragments are observed, each showing a characteristic blockage current, dwell time and structure-specific event signature (for the mtDNA).

References:

(1) Rooney, J. P.; Ryde, I. T.; Sanders, L. H.; Howlett, E. V.; Colton, M. D.; Germ, K. E.; Mayer, G. D.; Timothy Greenamyre, J.; Meyer, J. N. PCR Based Determination of Mitochondrial DNA Copy Number in Multiple Species. *Methods in Molecular Biology* **2015**, *1241*, 23–38.

(2) Khozhukhar, N.; Spadafora, D.; Rodriguez, Y.; Alexeyev, M. Elimination of Mitochondrial DNA from Mammalian Cells. *Curr Protoc Cell Biol* **2018**, *78* (1), 20.11.1-20.11.14. https://doi.org/10.1002/CPCB.39.
